# *Ganoderma lucidum*-Derived Meroterpenoids Show Anti-Inflammatory Activity In Vitro

**DOI:** 10.3390/molecules29051149

**Published:** 2024-03-05

**Authors:** Yun-Yun Liu, Dan Cai, Xin-Ping Tang, Yong-Xian Cheng

**Affiliations:** 1 State Key Laboratory of Southwestern Chinese Medicine Resources, School of Pharmacy, Chengdu University of Traditional Chinese Medicine, Chengdu 611137, China; 2 Institute for Inheritance-Based Innovation of Chinese Medicine, School of Pharmacy, Shenzhen University Medical School, Shenzhen University, Shenzhen 518055, China; 3 Marshall Laboratory of Biomedical Engineering, Shenzhen University Medical School, Shenzhen University, Shenzhen 518055, China

**Keywords:** *Ganoderma lucidum*, meroterpenoids, anti-inflammatory activities

## Abstract

*Ganoderma lucidum*, known as the “herb of spiritual potency”, is used for the treatment and prevention of various diseases, but the responsible constituents for its therapeutic effects are largely unknown. For the purpose of obtaining insight into the chemical and biological profiling of meroterpenoids in *G. lucidum*, various chromatographic approaches were utilized for the title fungus. As a result, six undescribed meroterpenoids, chizhienes A–F (**1**–**6**), containing two pairs of enantiomers (**4** and **5**), were isolated. Their structures were identified using spectroscopic and computational methods. In addition, the anti-inflammatory activities of all the isolates were evaluated by Western blot analysis in LPS-induced macrophage cells (RAW264.7), showing that **1** and **3** could dose dependently inhibit iNOS but not COX-2 expression. Further, **1** and **3** were found to inhibit nitric oxide (NO) production using the Greiss reagent test. The current study will aid in enriching the structural and biological diversity of *Ganoderma*-derived meroterpenoids.

## 1. Introduction

**Figure 1:**
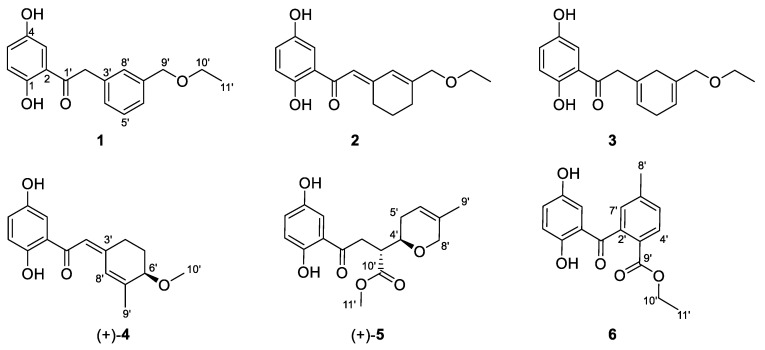
The structures of compounds **1**–**6**.

*Ganoderma lucidum* is distributed in all continents of the world, mostly growing in tropical, subtropical, and temperate regions [1], and has been used as a functional food based on traditional medicine for health and longevity in China and Southeast Asia for thousands of years [2]. Daily diets are commonly supplemented with *G. lucidum* to enhance their nutritional value, providing benefits such as improved serum mineral composition and bone index [3]. The fungus, also known as the “herb of spiritual potency”, was recorded in Sheng Nong’s Herbal Classic for the treatment and prevention of various diseases, such as neurasthenia, insomnia, anorexia, dizziness, chronic hepatitis, bronchitis, arthritis, nephritis, coronary heart disease, hypercholesterolemia, diabetes, hypertension, and cancer [4,5,6,7]. Previous phytochemical studies on this fungus focused on its polysaccharide and triterpenoid content. In addition, some other constituents such as alkaloids and sterols were also revealed [2,5]. *Ganoderma* meroterpenoids (GMs), the third most studied component with over 680 meroterpenoids beyond polysaccharides and triterpenoids, were first obtained in 2000 and have gained further attention since 2013, when lingzhiols were reported [8,9,10,11,12,13,14,15]. The basic structure of GMs is formed of two units: (A) a *p*-dihydroxybenzene and (B) a terpene moiety. These basic structure moieties could be independent or attached to another GM or other categories of structures to form structurally diverse GMs [8]. According to the number of carbon atoms contained in their side chains and the combinations with different additional moieties, GMs are divided into six categories: (1) ones with two isoprene units as side chains; (2) ones with three isoprene units as side chains; (3) dimers; (4) meroterpenoid–*p*-coumaric acid hybrids; (5) meroterpenoid–triterpenoid hybrids; and (6) meroterpenoid alkaloids [8]. Some members of the GMs possess biological properties such as antimicrobial [9,16], anti-allergic [17], renal-protective [18], antioxidant [19,20,21,22], antidiabetic [23,24], obesity-inhibitory [25], T-type calcium channel-blocking [26], 3-hydroxy-3-methyl glutaryl coenzyme A (HMG-CoA) reductase-inhibitory, α-glucosidase-inhibitory [27], acetylcholinesterase (AChE)-inhibitory [28], inflammation-suppressive [29,30], and tumor-suppressive activities [22], whereas the action mechanisms involved in GMs mainly focus on their inhibition of inflammation, tumors, and diabetes [31,32,33]. The current work is an in-depth investigation of *G*. *lucidum*, which led to the characterization of six meroterpenoids chizhienes A–F (**1**–**6**) (Figure 1). In addition, the anti-inflammatory effects of all the isolates were determined. All these efforts will be described below.

## 2. Results and Discussion

### 2.1. Structure Elucidation of the Compounds

**Figure 2:**
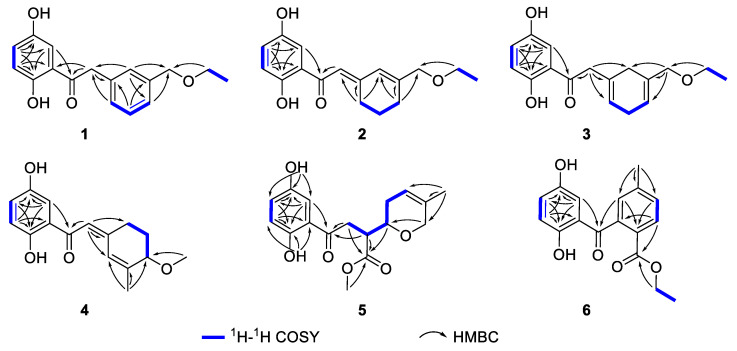
Key ^1^H-^1^H COSY and HMBC correlations for **1**–**6**.

Chizhiene A (**1**) was obtained as a yellow oil. The ^1^H NMR spectrum (Table 1) of 1 contains a typical ABX spin system [*δ*_H_ 7.34 (1H, d, *J* = 2.9 Hz, H-3), *δ*_H_ 7.00 (1H, dd, *J* = 8.9, 2.9 Hz, H-5), and *δ*_H_ 6.80 (1H, d, *J* = 8.9 Hz, H-6)]. The ^13^C and DEPT NMR spectra (Table 1) of **1** show one methyl, three methylenes (two oxygenated), seven aromatic methines, and six nonprotonated carbons (five aromatic and one ketone carbonyl at *δ*_C_ 205.3). The 1D NMR signals (Table 1) show high similarity to those of petchiene E [34], except for the presence of two additional methylenes at *δ*_C_ 73.5 (C-9′) and *δ*_C_ 66.8 (C-10′), one methyl at *δ*_C_ 15.4 (C-11′), and the absence of one ketone at *δ*_C_ 170 (C-9′) in **1**. These signals indicated that a methoxyethane group is located at C-7′ (*δ*_C_ 140.2) in **1** rather than a carboxylic acid group in petchiene E. The ^1^H-^1^H COSY correlation of H_3_-11′ (*δ*_H_ 1.20)/H_2_-10′ (*δ*_H_ 3.54) and the HMBC correlations of H_2_-10′, H-6′, and H-8′/C-9′ (Figure 2) supported the differences between **1** and petchiene E. Thus, the structure of **1** was identified.

Chizhiene B (**2**) was isolated as a yellow oil. Comparing the 1D and 2D NMR data (Table 1) of **2** and petchiene B [34] suggested that **2** is a structural analog of petchiene B. The difference between **2** and petchiene B is that the hydroxyl group at C-9′ in petchiene B is replaced by an ethoxy moiety in **2**. The ^1^H-^1^H COSY correlation of H_3_-11′/H_2_-10′ and the HMBC correlations of H_2_-10′/C-9′ and H_2_-6′/C-7′, C-8′, and C-9′ (Figure 2) confirmed the aforementioned conclusion. Thus, the structure of **2** was identified.

Chizhiene E (**3**) was obtained as yellow solids. The ^1^H NMR spectrum of **3** (Table 1) contains three typical aromatic signals at *δ*_H_ 7.30 (1H, d, *J* = 2.9 Hz, H-3), 7.01 (1H, dd, *J* = 8.9, 2.9 Hz, H-5), and 6.79 (1H, d, *J* = 8.9 Hz, H-6), indicating the presence of a 1,2,4-trisubstituted benzene substructure. The ^13^C NMR and DEPT spectra (Table 1) display 17 carbons classified into one methyl, five methylenes, five olefinic methines, and six nonprotonated carbons (one ketone carbonyl at *δ*_C_ 206.0). These NMR signals resemble those of **2** indicating they are analogs. Compound **3** differs from **2** only in the position of two double bonds. The Δ^3′(4′)^ and Δ^6′(7′)^ double bonds in **3** rather than the Δ^2′(3′)^ and Δ^7′(8′)^ in **2** were observed by the ^1^H-^1^H COSY correlations of H-4′ (*δ*_H_ 5.62)/H_2_-5′/H-6′ (*δ*_H_ 5.70) and HMBC correlations of H_2_-2′/C-1′, C-3′, C-4′, and C-8′ and H_2_-9′/C-6′, C-7′, and C-8′ (Figure 2). Therefore, the structure of **3** was assigned.

**Figure 3:**
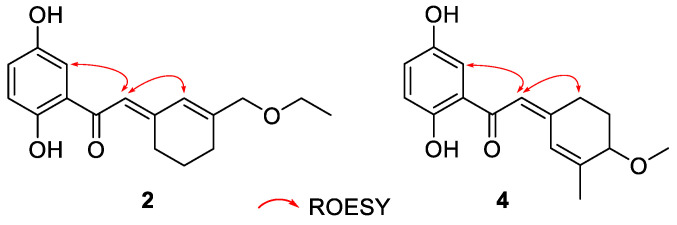
Key ROESY correlations for **2** and **4**.

**Figure 4:**
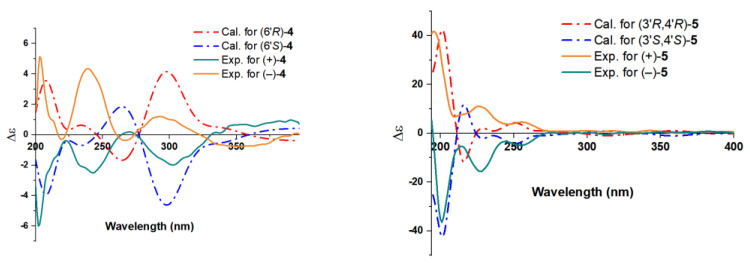
The calculated and experimental ECD spectra of **4** and **5**.

Chizhiene F (**4**) was afforded as yellow solids. A careful comparison of the NMR data of **4** with those of baoslingzhine C [35] indicated they are almost identical. The only difference between them is that the ethoxy group at C-6′ in baoslingzhine C is replaced by one methylol group in **4**, which was confirmed by ^1^H-^1^H COSY correlations of H_2_-4′/H_2_-5′/H-6′ (*δ*_H_ 3.86) and HMBC correlations of H_3_-9′/C-6′, C-7′, and C-8′ and H_3_-10′/C-6′ (Figure 2). Likewise, the *Z*-form of Δ^2′(3′)^ double bond is secured by ROESY correlations (Figure 3) of H-2′/H_2_-4′/H-3. There is one chiral center in **4**, and chiral HPLC analysis indicated that it is a racemate, whose chiral HPLC separation yielded (+)-**4** and (–)-**4** (Figure S49). To clarify the absolute configuration of each enantiomer, electronic circular diochroism (ECD) calculations were carried out and found that the calculated ECD spectrum of 6′*R*-**4** correlates well with the experimental one of (+)-**4**, enabling assigning the absolute configurations of (+)-**4** as 6′*R* and (–)-**4** as 6′*S* (Figure 4). The structure of **4** was therefore identified.

Chizhiene C (**5**) was collected as yellow solids. A detailed comparison of the ^1^H and ^13^C NMR data (Table 2) for compound **5** and lingzhine E [36] found that their similarity, except for an additional signal of the oxygenated methyl proton appeared as “s” (*δ*_H_ 3.60) and the oxygenated methyl carbon (*δ*_C_ 51.7) in **5**. It revealed that the former is the C-10′ methyl ester derivative of the latter. This hypothesis was verified by the key HMBC correlation of H_3_-11′/C-10′ (Figure 2). The observed coupling constant between H-3′/H-4′ (*J* = 5.9 Hz) enabled the assignment of **5** as the *threo* configurations [37]. **5** was subjected to chiral HPLC analysis followed by separation to yield its stereoisomers (Figure S50). Finally, the absolute configurations at the stereogenic centers of each isomer were, respectively, assigned as 3′*R*,4′*R* for (+)-**5** and 3′*S*,4′*S* for (–)-**5** using the same computational methods as that of **4** (Figure 4). The structure of **5** was therefore identified.

Chizhiene D (**6**) was obtained as yellow solids, presented the molecular formula C_17_H_16_O_5_ as inferred from the HRESIMS ion peak at *m*/*z* 323.0893 [M + Na]^+^ (calcd for C_17_H_16_O_5_Na^+^, 323.0890), ^13^C NMR, and DEPT spectra. The ^1^H NMR spectrum of **6** (Table 2) contains three typical aromatic signals at *δ*_H_ 6.47 (1H, d, *J* = 3.0 Hz, H-3), 7.00 (1H, dd, *J* = 8.9, 3.0 Hz, H-5), and 6.87 (1H, d, *J* = 8.9 Hz, H-6), indicating the presence of a 1,2,4-trisubstituted benzene substructure. The other three aromatic signals at *δ*_H_ 7.98 (1H, d, *J* = 8.0 Hz, H-4′), 7.48 (1H, br d, *J* = 8.0 Hz, H-5′), and 6.87 (1H, br s, H-7′) indicated the presence of the second 1,2,4-trisubstituted benzene substructure. The ^13^C NMR and DEPT spectra (Table 2) contain 17 resonances attributable to two methyls, one oxygenated methylene, six olefinic methines, and eight non-protonated carbons (one ester carbonyl at *δ*_C_ 167.0 and one ketone carbonyl at *δ*_C_ 204.2). The structure of **6** was mainly constructed assisted by 2D NMR data. The ^1^H-^1^H COSY correlation between H-5/H-6 and HMBC correlations between H-3/C-1 (*δ*_C_ 156.9), C-2, C-4 (*δ*_C_ 150.5), and C-1′ (*δ*_C_ 204.2) (Figure 2), considering the carbon chemical shifts of C-1 and C-4, suggested the presence of a 2,5-dihydroxybenzoyl moiety in **6**. The ^1^H-^1^H COSY correlation between H-4′/H-5′, in conjunction with HMBC correlations between H_3_-8′/C-5′ (*δ*_C_ 131.6), C-6′ (*δ*_C_ 145.2), and C-7′ (*δ*_C_ 129.0), H-5′, and H-7′/C-3′ (*δ*_C_ 127.3), H-4′/C-2′ (*δ*_C_ 141.6) and H-7′/C-1′ (Figure 2) strongly support the presence of an additional 1,2,4-trisubstituted benzene ring containing a methyl group connected to C-1′. The ethoxycarbonyl group is linked to C-3′ was supported by ^1^H-^1^H COSY correlation of H_2_-10′/H_3_-11′ and the HMBC correlations between H-4′, and H_2_-10′/C-9′. The structure of **6** was thus identified.

Of note, compounds **1**–**3** and **6** were found to bear an ethyl group in the structure, forming an ethoxy group. Since the structure for **2** without the ethyl group was characterized, ref. [34] and ethanol was used for extraction under heat, we highly speculate that all these isolates with the ethyl group should be artifacts during extraction procedures, although no further efforts were made to detect whether they are natural products or artifacts due to the extremely low content in the material. Further literature search found that compounds **1**, **3** and **6** and their ethyl products are undescribed, meaning that the structures of **1**, **3** and **6** with a terminal “OH” group are new natural products which will add structure diversity for GMs family. Despite the possible artifact nature for these compounds, the following biological potency for **1** and **3** may arise from the presence of the additional ethyl group, although further comparison between “-OH” and “CH_3_CH_2_-” forms was not conducted due to the unavailable amounts of the samples.

### 2.2. Biological Activity toward Inflammation

**Figure 5:**
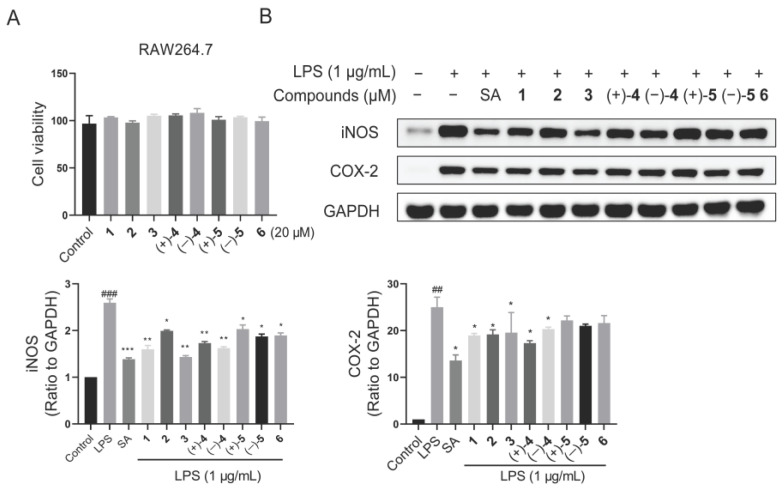
Anti-inflammatory effects of compounds **1**–**6** in LPS-induced RAW264.7 cells. (**A**) Cytotoxic effects of compounds **1**–**6** at 20 μM were examined in RAW264.7 cells. (**B**) Protein expression of iNOS and COX-2 at indicated concentrations of compounds and LPS for 24 h. ^##^ *p* < 0.01 versus control group; ^###^ *p* < 0.001 versus control group; * *p* < 0.05 versus LPS group; ** *p* < 0.01 versus LPS group; and *** *p* < 0.001 versus LPS group. One-way ANOVA. Data are represented as the mean ± SEM. LPS: lipopolysaccharide. SA: sappanone A.

Inflammation is an essential process that allows our bodies to fight against various pathogenic bacteria, viruses, and parasites [38]. The production of nitric oxide (NO) and the expression of inducible nitric oxide synthase (iNOS) and cyclooxygenase-2 (COX-2) proteins are tightly associated with inflammation, indicating its occurrence to a certain extent. *Ganoderma* fungi have been reported to have anti-inflammatory effects [39]. Therefore, the anti-inflammatory activities of all the isolates were evaluated. Initially, the cytotoxic effects of compounds were assessed using the cell proliferation and toxicity detection kit (CCK8) assay. As shown in Figure 5, there was no cytotoxicity of compounds observed in RAW264.7 cells at 20 μM for 24 h. 

**Figure 6:**
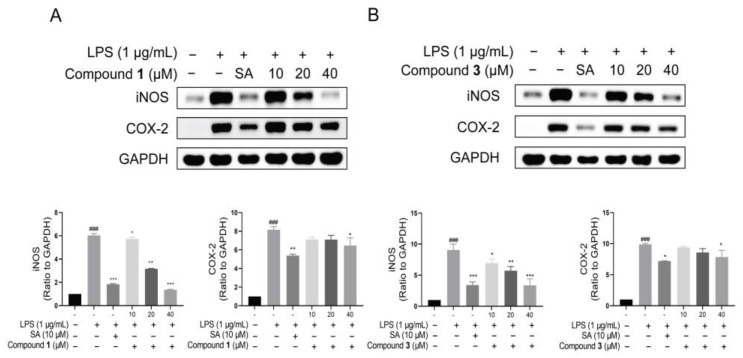
Effects of compounds **1** and **3** on the protein levels of iNOS and COX-2. RAW 264.7 cells were treated with different concentrations of compounds **1** and **3** (10, 20 and 40 μM). (**A**,**B**) Compounds **1** and **3** down regulate protein expression of iNOS in a dose-dependent manner. ^###^ *p* < 0.001 versus control group; * *p* < 0.05 versus LPS group; ** *p* < 0.01 versus LPS group; and *** *p* < 0.001 versus LPS group. One-way ANOVA. Data are represented as the mean ± SEM. LPS: lipopolysaccharide. SA: sappanone A.

**Figure 7:**
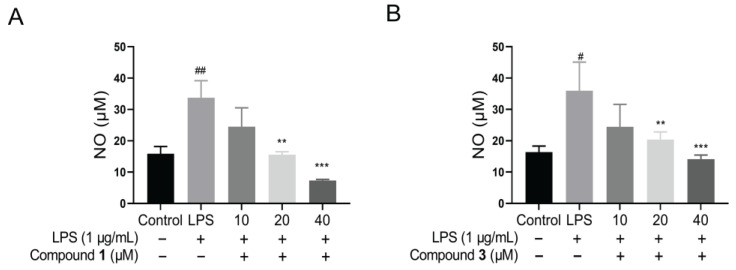
The effect of NO release of compounds **1** and **3** in LPS-stimulated RAW264.7 cells. (**A**,**B**) NO production inhibition in RAW 264.7 cells, treated with compounds **1** and **3** (10, 20 and 40 μM). ^#^ *p* < 0.05 versus control group; ^##^ *p* < 0.01 versus control group; ** *p* < 0.01 versus LPS group. LPS; *** *p* < 0.001 versus LPS group. One-way ANOVA. Data are represented as the mean ± SEM. LPS: lipopolysaccharide.

Following this, the protein expression of iNOS and COX-2 was detected by the Western blotting assay in LPS-stimulated macrophage RAW264.7 cells. The results revealed that all compounds down regulated iNOS protein, particularly compounds **1** and **3** (Figure 5). Hence, a dose–response curve for compounds **1** and **3** was further performed. Similarly, the cytotoxic effects of compounds **1** and **3** were firstly detected. The results showed that no cytotoxicity for compound **1** and faint cytotoxicity for compound **3** at 40 μM (Figure S53). Then, the Western blotting assay revealed that the protein level of iNOS was down regulated by compounds **1** and **3** dose dependently in LPS-induced RAW264.7 cells (Figure 6). Meanwhile, the NO production of compounds **1** and **3** was also examined. It was found that compounds **1** and **3** both could inhibit NO release in LPS-stimulated RAW264.7 cells (Figure 7). Interestingly, compounds **1** and **3**, rather than **2** and **4**, are active toward inflammation inhibition. Upon inspecting their structures, we could conclude that the Δ^2′(3′)^ double bond might have an influence on the biological activity. In detail, the presence of the Δ^2′(3′)^ double bond is not advantageous for keeping the anti-inflammatory property. These findings may provide inspirations for structure optimization using these meroterpenoids as lead compounds against inflammation. 

## 3. Experimental Section

### 3.1. General Procedures

Optical rotations were measured on an Anton Paar MCP 100 polarimeter. A JASCO J-815 CD spectrometer was employed for recording UV and CD spectra of **2**, **4**, and **5**. UV spectra of **1**, **3**, and **6** were collected on a GENESYS 150 uv-visible spectrophotometer. The 1D and 2D NMR data of **1**–**6** were obtained on a Bruker AV-600 MHz spectrometer with TMS as an internal standard. The HRESIMS data of **1**–**6** were carried out on a SCIEX X500R QTOF MS spectrometer. Sephadex LH-20 (Amersham Pharmacia, Uppsala, Sweden), reversed-phase C-18 silica gel (40–60 μm; Daiso Co., Osaka, Japan) and MCI gel CHP 20P (75–150 μm, Mitsubishi Chemical Industries, Tokyo, Japan) were used for column chromatography (CC). For semi-preparative HPLC, a Saipuruisi (LC-52, SEP, Beijing, China) chromatograph with a COSMOSIL column (5C_18_-MS-II, 10 mm i.d. × 250 mm) was carried out. Chiral HPLC analysis were conducted on an Agilent technologies 1260 infinity II liquid chromatograph using Daicel Chiralpak IC (250 mm × 10 mm, i.d., 5 μm) chiral columns.

### 3.2. Fungal Material

The source and authentication of *G. lucidum* fruiting bodies were identical with our previous study [35] and the voucher specimen (CHYX-0619) of *G. lucidum* has been deposited in Inheritance-Based Innovation of Chinese Medicine, School of Pharmacy, Shenzhen University Medical School, Shenzhen University.

### 3.3. Extraction and Isolation

The initial extraction process of the dried fruiting bodies of *G. lucidum* (500.0 kg) and fractionation of the extract to yield 17 fractions (Fr.1–Fr.17) refers to a previous report [35].

Fr.11 (379.0 g) was cut into six parts (Fr.11.1–Fr.11.6) over an MCI gel CHP 20P column (aqueous MeOH, 30–100%). And then, Fr.11.1 (23.4 g) was separated by using Sephadex LH-20 to afford five portions (Fr.11.1.1–Fr.11.1.5). Among them, the second part of the fraction Fr.11.1 (1.6 g) was applied to RP-18 CC (MeOH/H_2_O, 55–100%) to give nine sub-fractions (Fr.11.1.2.1–Fr.11.1.2.9). Fr.11.1.2.6 (305.5 mg) was eluted on preparative TLC (CH_2_Cl_2_–acetone, 15:1) to obtain Fr.11.1.2.6.1–Fr.11.1.2.6.6 from their respective TLC bands. The third band Fr.11.1.2.6.3 (15.7 mg) and the fourth band Fr.11.1.2.6.4 (74.8 mg) were, respectively, purified by semi-preparative HPLC to yield **6** (0.94 mg, MeCN/H_2_O, 38%, 3.0 mL/min, *t*_R_ = 54.28 min) and **1** (3.78 mg, MeCN/H_2_O, 43%, 3.0 mL/min, *t*_R_ = 29.03 min). Fr.11.2 (67.2 g) was divided into four portions (Fr.11.2.1–Fr.11.2.4) by using Sephadex LH-20. Among them, the third part of the fraction Fr.11.1 (1.6 g) was further fractionated by using RP-18 CC (MeOH/H_2_O, 40–100%), yielding fifteen sub-fractions (Fr.11.2.3.1–Fr.11.2.3.15). And then Fr.11.2.3.6 (1.0 g) and Fr.11.2.3.7 (1.6 g) were, respectively, passed through Sephadex LH-20 to produce seven fractions (Fr.11.2.3.6.1–Fr.11.2.3.6.3 from the former and Fr.11.2.3.7.1–Fr.11.2.3.7.4 from the latter). Fr.11.2.3.6.2 was subjected to Sephadex LH-20 to produce four fractions Fr.11.2.3.6.2.1–Fr.11.2.3.6.2.4. And then, Fr.11.2.3.6.2.4 (84.0 mg), Fr.11.2.3.6.3 (62.5 mg), and Fr.11.2.3.7.2 (128.6 mg) were further refined by semi-preparative HPLC to afford **5** (4.67 mg, *t*_R_ = 17.53 min, MeOH/H_2_O, 66%, 3.0 mL/min), **4** (1.26 mg, *t*_R_ = 44.95 min, MeCN/H_2_O, 38%, 3.0 mL/min) and **3** (1.58 mg, *t*_R_ = 15.82 min, MeOH/H_2_O, 67%, 3.0 mL/min), respectively. 

Fr.13 (736.0 g) was submitted to MCI gel CHP 20P CC for further fractionation, eluted with aqueous MeOH (65–100%) to generate eight fractions (Fr.13.1–Fr.13.8). Among them, Fr.13.4 (116.3 g) was separated by means of Sephadex LH-20 to afford three portions (Fr.13.4.1–13.4.3). And then Fr.13.4.2 (1.9 g) was passed through Sephadex LH-20 gel to produce four fractions (Fr.13.4.2.1–Fr.13.4.2.4). Fr.13.4.2.2 (998.6 mg) was divided into twelve parts (Fr.13.4.2.2.1–Fr.13.4.2.2.12) by gradient elution of RP-18 CC (aqueous MeOH, 40–100%). Among them, the seventh sub-fraction (Fr.13.4.2.2.7, 135.8 mg) was further separated using semi-preparative HPLC to give **2** (4.69 mg, *t*_R_ = 30.71 min, MeOH/H_2_O, 67%, 3.0 mL/min).

Compounds **4** and **5** were found to be racemic by analysis of chiral phase HPLC on Daicel Chiralpak IC column. Subsequently, two racemic mixtures were purified by chiral phase HPLC on same column (*n*-hexane/ethanol, 95:5), respectively, to give enantiomers (+)-**4** (0.55 mg, *t*_R_ = 20.48 min) and (–)-**4** (0.50 mg, *t*_R_ = 26.81 min); (+)-**5** (2.1 mg, *t*_R_ = 23.98 min) and (–)-**5** (1.8 mg, *t*_R_ = 26.18 min).

### 3.4. Compound Characterization Data

*Chizhiene A* (**1**): yellow oils. UV (MeOH) *λ*_max_ (log*ε*) 365 (3.41), 257 (3.71), 212 (4.10) nm; HRESIMS: *m*/*z* 309.1097 [M + Na]^+^ (calcd for C_17_H_18_O_4_Na^+^, 309.1097); ^1^H and ^13^C NMR data; see Table 1.

*Chizhiene B* (**2**): yellow oils. UV (MeOH) *λ*_max_ (log*ε*) 385 (3.50), 317 (4.02), 228 (3.78) nm; HRESIMS: *m*/*z* 289.1432 [M + H]^+^ (calcd *for* C_17_H_21_O_4_^+^, 289.1434); ^1^H and ^13^C NMR data; see Table 1.

*Chizhiene E* (**3**): yellow solids. UV (MeOH) *λ*_max_ (log*ε*) 365 (3.36), 257 (3.61), 213 (3.96) nm; HRESIMS: *m*/*z* 311.1254 [M + Na]^+^ (calcd *for* C_17_H_20_O_4_Na^+^, 311.1254); ^1^H and ^13^C NMR data; see Table 1.

*Chizhiene F* (**4**): yellow *solids*. [*α*]_D_^20^ +3.6 (*c* 0.28, MeOH), (+)-**4**; [*α*]_D_^20^ –14.3 (*c* 0.21, MeOH), (–)-**4**; UV (MeOH) *λ*_max_ (log*ε*) 319 (4.10), 227 (3.95) nm; HRESIMS: *m*/*z* 275.1278 [M + H]^+^ (calcd. for C_16_H_19_O_4_^+^, 275.1278); ^1^H and ^13^C NMR data; see Table 2.

*Chizhiene C* (**5**): yellow solids. [*α*]_D_^25^ +75.0 (*c* 0.32, MeOH), (+)-**5**; [*α*]_D_^25^ –57.7 (*c* 0.26, MeOH), (–)-**5**; UV (MeOH) *λ*_max_ (log*ε*) 364 (4.01), 256 (4.24), 227 (4.54) nm; HRESIMS: *m*/*z* 321.1331 [M + H]^+^ (calcd. for C_17_H_21_O_6_^+^, 321.1333); ^1^H and ^13^C NMR data; see Table 2.

*Chizhiene D* (**6**): yellow solids. UV (MeOH) *λ*_max_ (log*ε*) 368 (3.63), 231 (4.37), 205 (4.50) nm; HRESIMS: *m*/*z* 323.0893 [M + Na]^+^ (calcd. for C_17_H_16_O_5_Na^+^, 323.0890); ^1^H and ^13^C NMR data; see Table 2.

### 3.5. ECD Calculations for Compounds **4** and **5**

To confirm the absolute configurations of the enantiomers of **4** and **5**, theoretical ECD spectra were calculated and compared with the corresponding experimental spectra. The predominant conformers of **4** and **5** were optimized with Gaussian 09 [40] at the B3LYP/6-311g(d,p) level. Subsequently, the optimized conformers were used for ECD calculations using the same method. Solvent effects were taken into account using the polarizable-conductor calculation model (PCM) with methanol as the solvent. The conclusion indicated that the ECD spectra of (6′*S*)-**4**, (6′*R*)-**4**, (3′*R*,4′*R*)-**5**, and (3′*S*,4′*S*)-**5** agree well with the experimental data of (+)-**4**, (–)-**4**, (+)-**5**, and (–)-**5** (Figure 4). 

### 3.6. Cell Culture

The murine macrophage cell line RAW264.7 (Cell Bank of China Science Academy, Shanghai, China) was grown in DMEM medium (DMEM High Glucose, C3113-0500, 2350408, Viva Cell, Shanghai, China), containing 10% FBS (Fetal Bovine Serum, 2364724, Gibco, Shanghai, America), 1% penicillin, and 1% streptomycin at 37 °C under 5% CO_2_ condition. 

### 3.7. Cell Viability Assay

The influence of the compounds on cell viability were evaluated by the CCK8 assay using a commercial kit (CCK-8, MA0218, MA0218-Jun-15I, MeilunBio, Dalian, China). The cells were seeded into 96-well culture plates (5 × 10^3^ cells/well) overnight and treated with compounds **1**–**6** (20 μM) for 24 h. Subsequently, CCK8 solution was incubated with cells for another 1 h, and OD 450 nm values were detected by Cytation1 (BioTek, Winooski, VT, USA).

### 3.8. Measurement of NO Production

RAW 264.7 cells were seeded in a 24-well plate at 1 × 10^5^ cells/well overnight and treated with compounds **1** and **3** (10 μM, 20 μM, and 40 μM) and LPS for 24 h. Cell culture medium was collected and mixed with equal volumes of Griess reagent (Nitric Oxide Assay Kit, S0021M, 042723230918 Beyotime, Shanghai, China) at room temperature in the dark [41]. The OD values were measured using Cytation1 (BioTek, Winooski, VT, USA) at 540 nm and NO production was detected using a sodium nitrite standard calibration curve.

### 3.9. Western Blotting Analysis

Western blotting assays were carried out as previous studies [42,43,44]. RAW264.7 cells were cultured with LPS (1 μg/mL) and indicated concentrations of compounds for 24 h, then, washed with pre-cold PBS and lysed using radio-immunoprecipitation assay buffer (RIPA, R6166S, 230330G02-01, US Everbright, Suzhou, China) containing proteinase (Protease Inhibitors, MIKX, DB612A-01, 23LB0817W, Shenzhen, China) and phosphatase inhibitors [Protein Phosphatase Inhibitor Complex (100×), MB12707, MeilunBio, Dalian, China]. Protein extracts were separated by 10% SDS-PAGE and transferred to PVDF membranes. Anti-iNOS (iNOS (D6B6S) Rabbit mAb #13120S, CST, Boston, MA, USA), anti-COX-2 (COX2 (D5H5) XP^®^ Rabbit mAb #12282S, CST, Boston, MA, USA), and anti-GAPDH (GAPDH (D16H11) XP^®^ Rabbit mAb #5174S, CST, Boston, MA, USA) antibodies were incubated with the PVDF membranes at 4 °C overnight after blocking with 5% nonfat milk. The horseradish peroxidase (HRP)-conjugated corresponding secondary antibodies were incubated with the PVDF membranes and a chemiluminescence detection kit (Fort Pierce, FL, USA) was used for detecting the bands. The ImageJ software (1.52a) was used to detect the density of immunoblot results.

### 3.10. Statistical Analysis 

All the experimental data were performed in three replicates. The results are represented as the mean ± SEM. Statistical analyses were carried out using GraphPad Prism 8 with Student’s *t*-test, and one-way ANOVA. Differences were considered significant with * *p* < 0.05 or ^#^ *p* < 0.05. 

## 4. Conclusions

To conclude, the current study led to the characterization of six new meroterpenoids (**1**–**6**) from *G. lucidum.* The possible artificial nature of compounds **1**–**3** and **6** and their contribution to biological potential were briefly discussed. Biological evaluations revealed that compounds **1** and **3** could significantly attenuate the protein expression level of iNOS and NO production in LPS-stimulated RAW264.7 cells, indicating their potential in inflammatory disease. In addition, the present findings are also beneficial for insights into GMs structure alterations in the context of trace content in the material. Last but not least, the hydroxy group on the benzene ring is more readily reacted with ethanol than a primary alcohol, contrary to our present observations. 

## Figures and Tables

**Table 1:** ^1^H (600 MHz) and ^13^C (150 MHz) NMR data of **1**–**3** (*δ* in ppm, *J* in Hz, in methanol-*d*_4_).

	1		2		3	
No.	*δ*_H_	*δ*_C_	*δ*_H_	*δ*_C_	*δ*_H_	*δ*_C_
1		157.1, C		157.3, C		156.9, C
2		120.2, C		122.2, C		120.4, C
3	7.34 (d, 2.9)	116.2, CH	7.25 (d, 2.9)	115.2, CH	7.30 (d, 2.9)	116.1, CH
4		150.6, C		150.4, C		150.6, C
5	7.00 (dd, 8.9, 2.9)	126.0, CH	6.97 (dd, 8.9, 2.9)	125.1, CH	7.01 (dd, 8.9, 2.9)	126.0, CH
6	6.80 (d, 8.9)	119.8, CH	6.77 (d, 8.9)	119.5, CH	6.79 (d, 8.9)	119.7, CH
1′		205.3, C		197.1, C		206.0, C
2′	4.31 (s)	46.2, CH_2_	6.76 (br s)	119.6, CH	3.68 (br s)	47.9, CH_2_
3′		136.2, C		157.3, C		130.7, C
4′	7.22 (br d, 7.6)	130.0, CH	3.00 (t-like, 6.3)	28.7, CH_2_	5.62 (m)	124.1, CH
5′	7.31 (t, 7.6)	129.7, CH	1.78 (p, 6.3)	23.2, CH_2_	2.77 (m)	28.4, CH_2_
6′	7.23 (br d, 7.6)	127.5, CH	2.21 (t, 6.3)	27.3, CH_2_	5.70 (m)	122.7, CH
7′		140.2, C		151.3, C		133.4, C
8′	7.28 (br s)	129.9, CH	6.36 (br s)	127.5, CH	Ha: 2.66 (d, 8.0)	31.2, CH_2_
					Hb: 2.65 (d, 8.0)	
9′	4.49 (s)	73.5, CH_2_	4.04 (br s)	74.7, CH_2_	3.88 (br s)	75.6, CH_2_
10′	3.54 (q, 7.0)	66.8, CH_2_	3.53 (q, 7.0)	67.1, CH_2_	3.45 (q, 7.0)	66.2, CH_2_
11′	1.20 (t, 7.0)	15.4, CH_3_	1.23 (t, 7.0)	15.5, CH_3_	1.17 (t, 7.0)	15.4, CH_3_

**Table 2:** ^1^H (600 MHz) and ^13^C (150 MHz) NMR data of **4** and **6** (in methanol-*d*_4_) and **5** (in DMSO–*d*_6_) (*δ* in ppm, *J* in Hz).

	4		5		6	
No.	*δ*_H_	*δ*_C_	*δ*_H_	*δ*_C_	*δ*_H_	*δ*_C_
1		157.4, C		153.2, C		156.9, C
2		122.1, C		120.4, C		121.4, C
3	7.24 (d, 2.9)	115.4, CH	7.20 (d, 3.0)	114.5, CH	6.47 (d, 3.0)	117.8, CH
4		150.4, C		149.5, C		150.5, C
5	6.97 (dd, 8.9, 2.9)	125.2, CH	6.98 (dd, 8.8, 3.0)	124.2, CH	7.00 (dd, 8.9, 3.0)	125.9, CH
6	6.78 (d, 8.9)	119.7, CH	6.81 (d, 8.8)	118.4, CH	6.87 (d, 8.9)	119.6, CH
1′		196.6, C		203.0, C		204.2, C
2′	6.64 (br s)	117.1, C	Ha: 3.35 (dd, 18.4, 3.6)	37.6, CH_2_		141.6, C
			Hb: 3.44 (dd, 18.4, 10.3)			
3′		154.9, C	3.03 (ddd, 10.3, 5.9, 3.6)	44.9, CH		127.3, C
4′	Ha: 2.66 (dddd, 15.4, 8.5, 4.0, 1.4)	30.4, CH_2_	3.70 (ddd, 10.1, 5.9, 3.4)	73.2, CH	7.98 (d, 8.0)	131.3, CH
	Hb: 2.45 (dddd, 15.4, 8.9, 4.0, 1.4)					
5′	Ha: 2.06 (ddt, 12.9, 8.5, 4.0)	28.4, CH_2_	Ha: 2.09 (m)	27.9, CH_2_	7.48 (br d, 8.0)	131.6, CH
	Hb: 1.90 (dddd, 12.9, 8.9, 6.4, 4.0)		Hb: 1.91 (br d, 17.2)			
6′	3.86 (t-like, 5.3)	78.9, CH	5.47 (m)	117.7, CH		145.2, C
7′		150.7, C		132.6, C	7.25 (br s)	129.0, CH
8′	7.41 (br s)	125.4, CH	3.96 (br s)	68.7, CH_2_	2.47 (br s)	21.5, CH_3_
9′	1.97 (br s)	21.6, CH_3_	1.56 (br s)	18.2, CH_3_		167.0, C
10′	3.44 (s)	57.5, CH_3_		173.0, C	4.11 (q, 7.1)	62.5, CH_2_
11′			3.60 (s)	51.7, CH_3_	1.10 (t, 7.1)	13.9, CH_3_
1-OH			10.91 (s)			
4-OH			9.17 (s)			

## Data Availability

All the data in this research are presented in manuscript and Supplementary Materials.

## Supplementary Materials

The following supporting information can be downloaded at: https://www.mdpi.com/article/10.3390/molecules29051149/s1. Figures S1–S8: NMR and UV spectra and HRESIMS of 1. Figures S9–S17: NMR and UV spectra and HRESIMS of 2. Figures S18–S24: NMR and UV spectra and HRESIMS of 3. Figures S25–S31: NMR spectra and HRESIMS of 4. Figures S32–S33: UV and CD spectra of (±)-4. Figures S34–S39: NMR spectra and HRESIMS of 5. Figures S40–S41: UV and CD spectra of (±)-5. Figures S42–S48 NMR and UV spectra and HRESIMS of 6. Figure S49: Chiral HPLC separation of racemic 4. Figure S50: Chiral HPLC separation of racemic 5. Figure S51: The lowest-energy conformers of 4. Figure S52: The lowest-energy conformers of 5. Figure S53: The cytotoxic effects of compounds 1 and 3 in RAW264.7 cells. Table S1: Extracted heats and weighting factors of the optimized conformers of 4 and 5. Table S2: The Cartesian coordinates of the lowest-energy conformers for 4 and 5.
