# Supplementary material for: Ganoderma lucidum-Derived Meroterpenoids Show Anti-Inflammatory Activity In Vitro
Source: Molecules. 2024 Mar 5;29(5):1149. doi: 10.3390/molecules29051149 (PMC10935275; doi:10.3390/molecules29051149)
Supplement: Supplementary file 1 [file molecules-29-01149-s001.zip › molecules-2882988-supplementary.pdf]

Ganoderma lucidum-Derived Meroterpenoids Show Anti-Inflammatory Activity In Vitro

Yun-Yun Liu [1,2,3], Dan Cai [2,3], Xin-Ping Tang [2,3] and Yong-Xian Cheng [1,2,3,*]

1 State Key Laboratory of Southwestern Chinese Medicine Resources, School of Pharmacy, Chengdu University of Traditional Chinese Medicine, Chengdu 611137, China

2 Institute for Inheritance-Based Innovation of Chinese Medicine, School of Pharmacy, Shenzhen University Medical School, Shenzhen University, Shenzhen 518055, China

3 Marshall Laboratory of Biomedical Engineering, Shenzhen University Medical School, Shenzhen University, Shenzhen 518055, China

* Correspondence: yxcheng@szu.edu.cn; Tel.: +86-0755-26902073

Table of Contents

1. **Figures S1–S8 NMR and UV spectra and HRESIMS of 1**
2. **Figures S9–S17 NMR and UV spectra and HRESIMS of 2**
3. **Figures S18–S24 NMR and UV spectra and HRESIMS of 3**
4. **Figures S25–S31 NMR spectra and HRESIMS of 4**
5. **Figures S32–S33 UV and CD spectra of (±)-4**
6. **Figures S34–S39 NMR spectra and HRESIMS of 5**
7. **Figures S40–S41 UV and CD spectra of (±)-5**
8. **Figures S42–S48 NMR and UV spectra and HRESIMS of 6**
9. **Figure S49. Chiral HPLC separation of racemic 4**
10. **Figure S50. Chiral HPLC separation of racemic 5**
11. **Figure S51. The lowest energy conformers of 4**
12. **Figure S52. The lowest energy conformers of 5**
13. **Figure S53. The cytotoxic effects of compounds 1 and 3 in RAW264.7 cells**
14. **Table S1. Extracted heats and weighting factors of the optimized conformers of 4 and 5**
15. **Table S2. The Cartesian coordinates of the lowest energy conformers for 4 and 5**

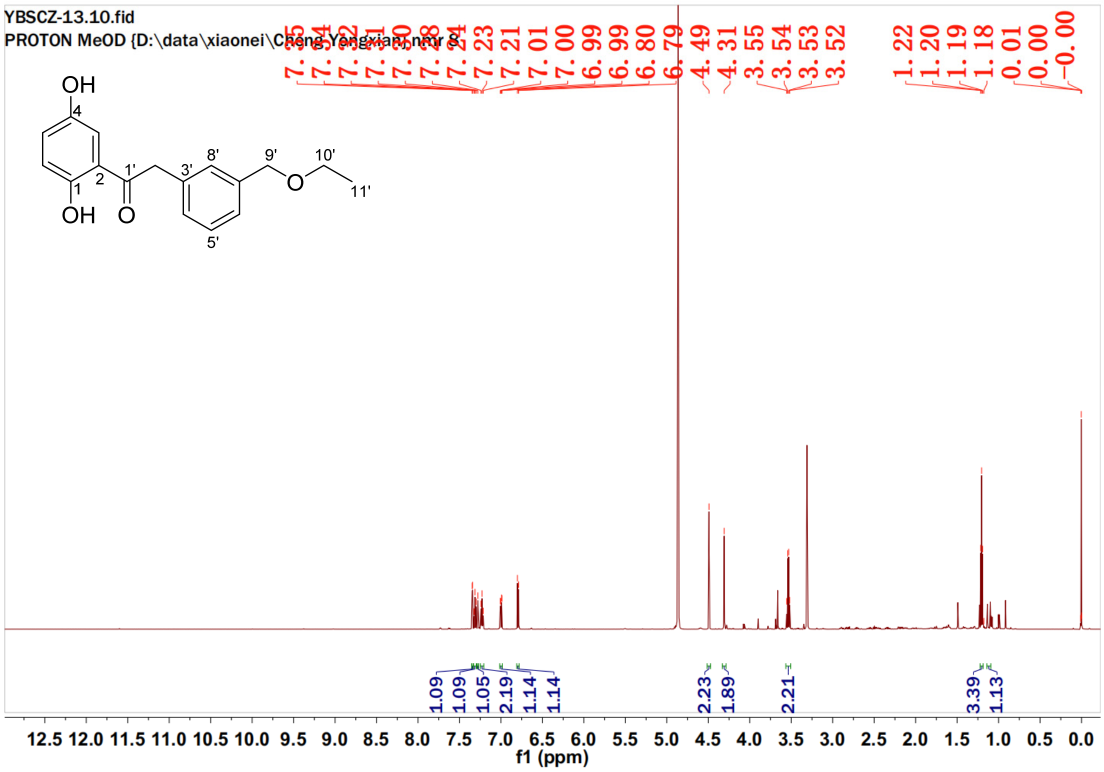

Figure S1. ^{1}H NMR spectrum of **1** in methanol-*d*4.

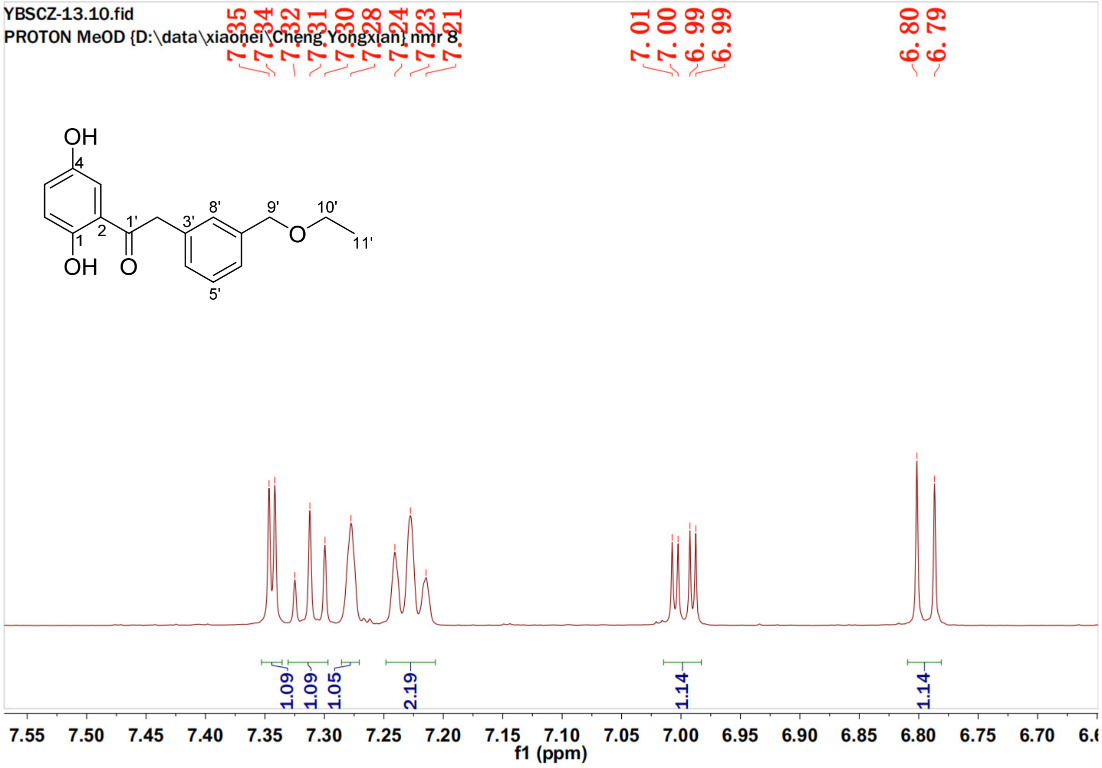

Figure S2. Enlarged ^{1}H NMR spectrum of **1** in methanol-*d*4.

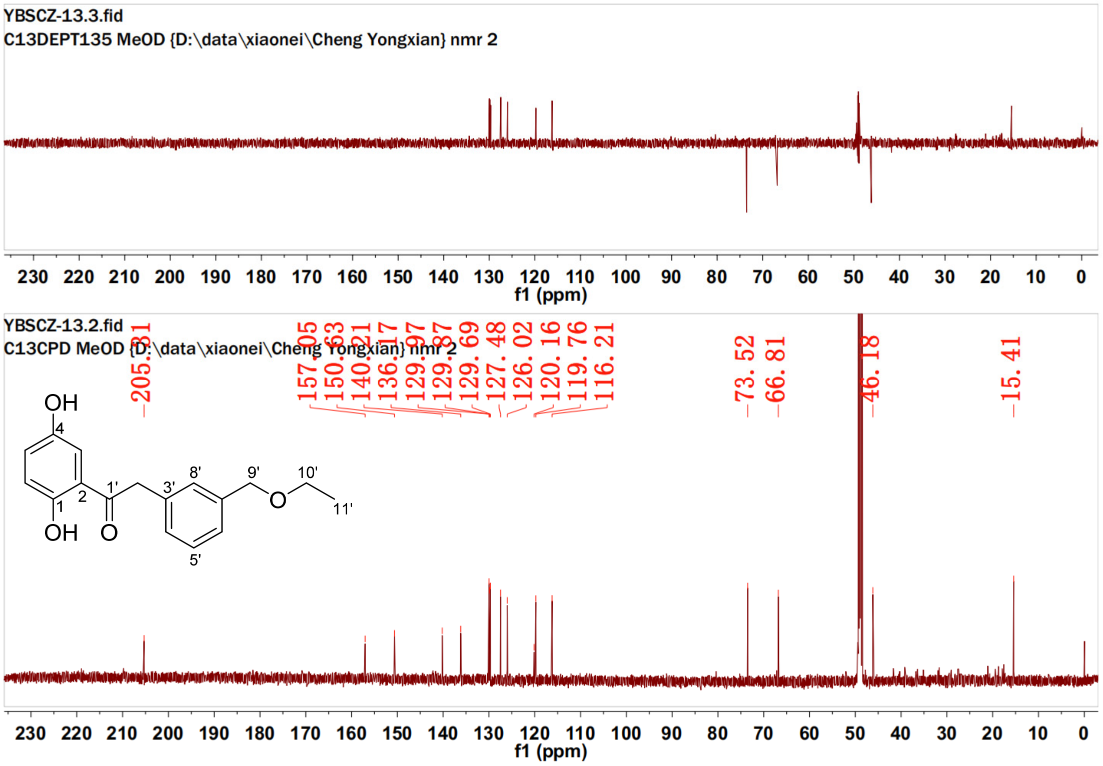

Figure S3. ^{13}C NMR and DEPT spectra of **1** in methanol-d_4.

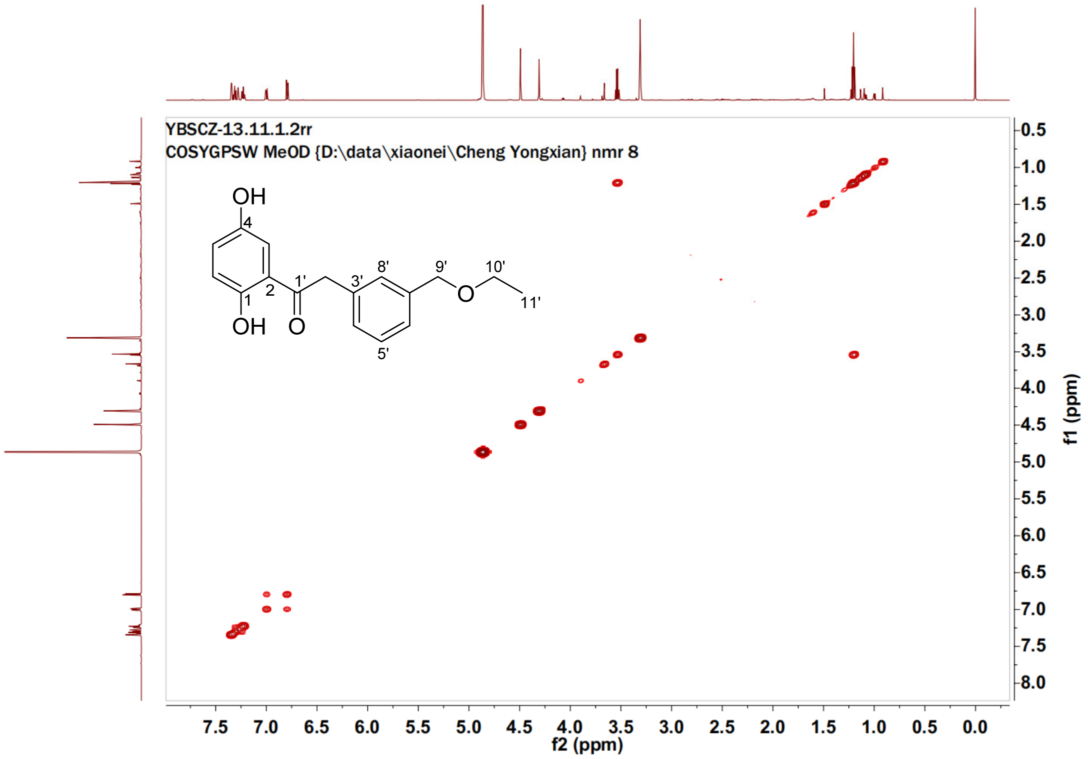

Figure S4. ^{1}H-^{1}H COSY spectrum of **1** in methanol-d_4.

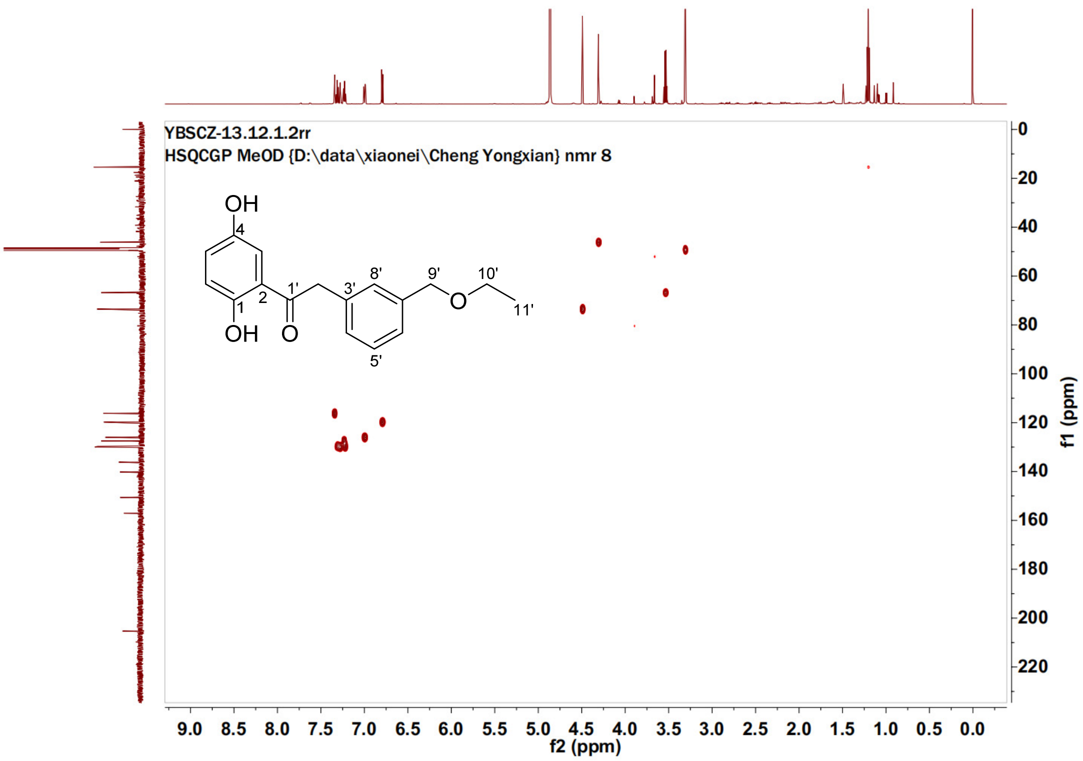

Figure S5. HSQC spectrum of **1** in methanol-d_4.

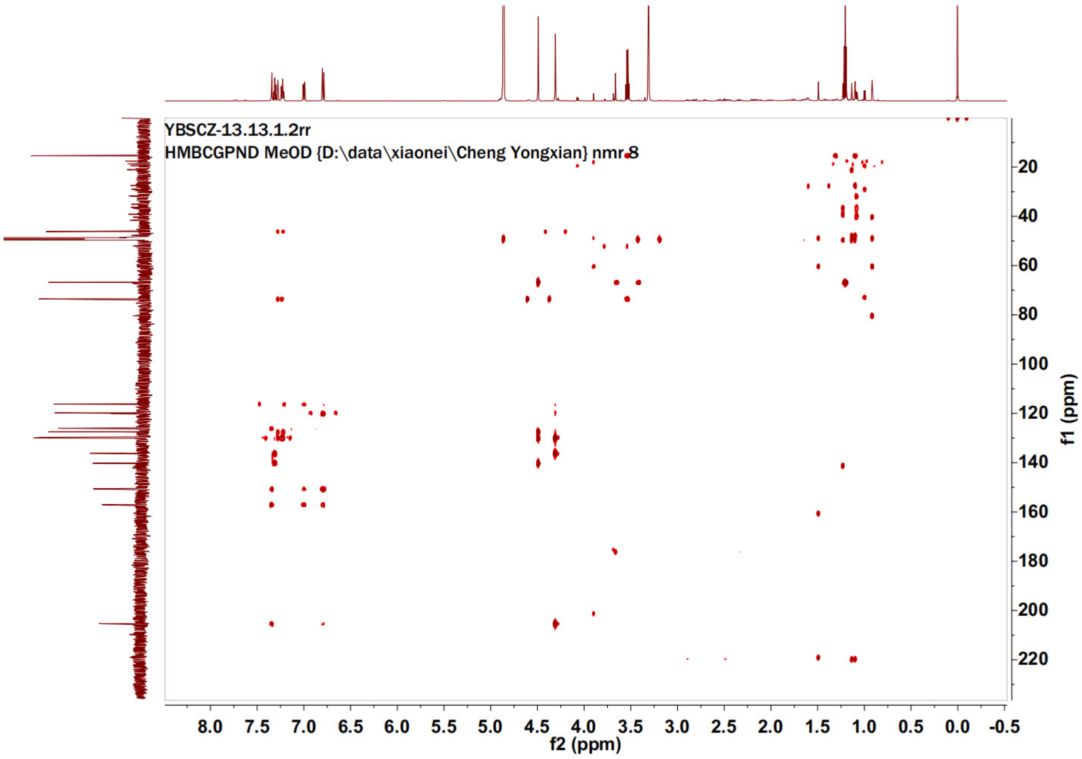

Figure S6. HMBC spectrum of **1** in methanol-d_4.

Range 190 - 450 nm

interval 1.0 nm

speed slow

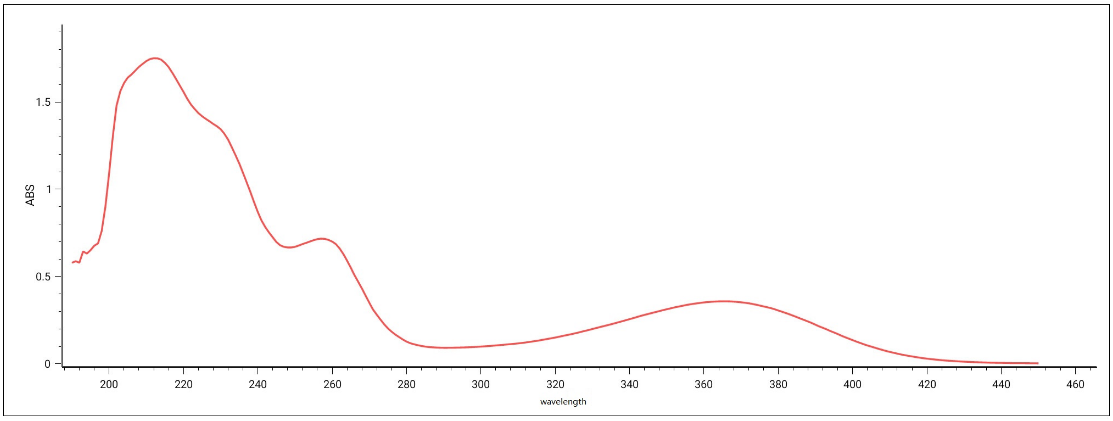

sample

sample 1

Figure S7. UV spectrum of **1**.

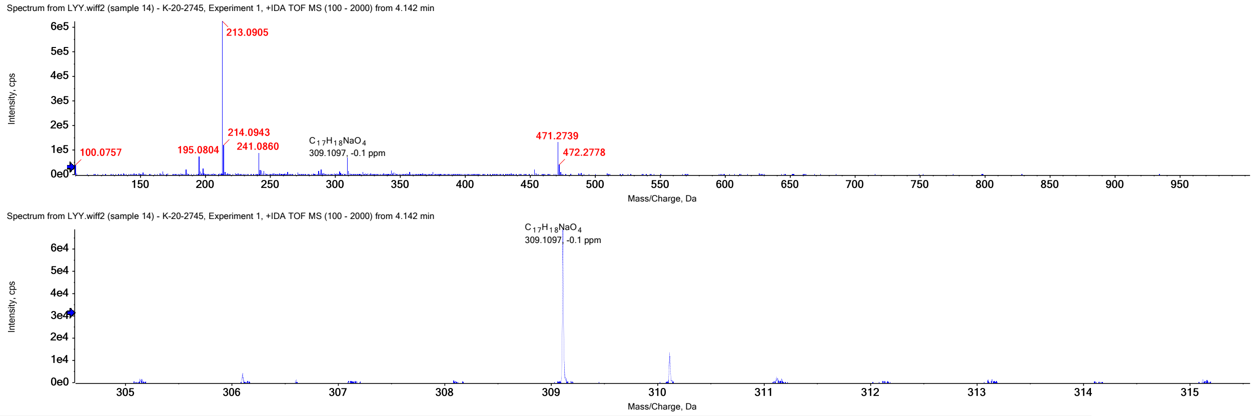

Hit	Formula	m/z	RDB	ppm	MS Rank	MSMS ppm	MSMS Rank	Found
1	C17H18O4	309.1097	9.0	-0.1	1			NA/NA

Figure S8. HRESIMS of **1.**

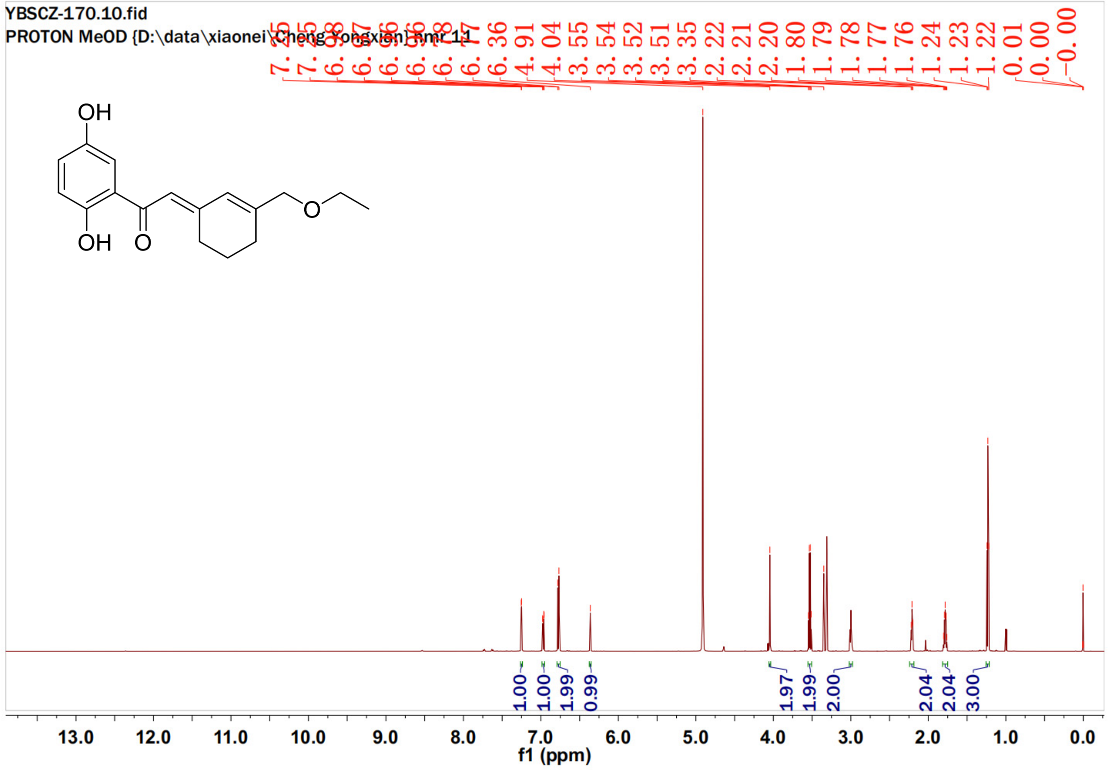

Figure S9. ^{1}H NMR spectrum of **2** in methanol-d_4.

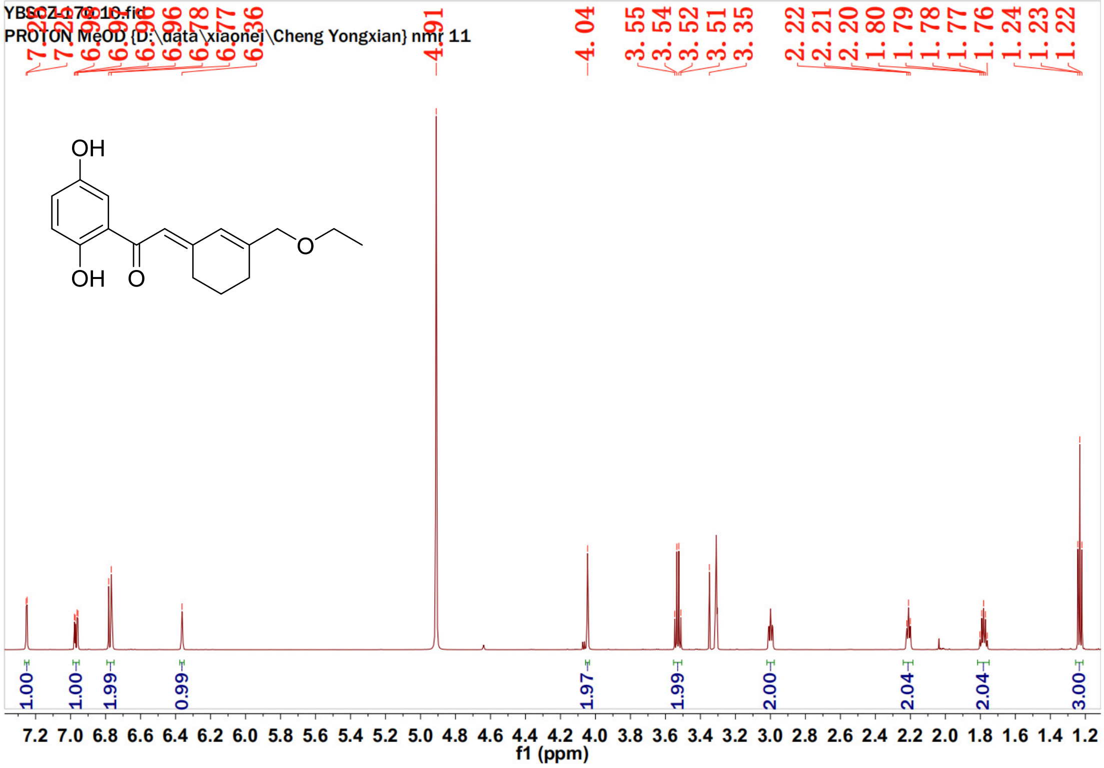

Figure S10. Enlarged ^{1}H NMR spectrum of **2** in methanol-d_4.

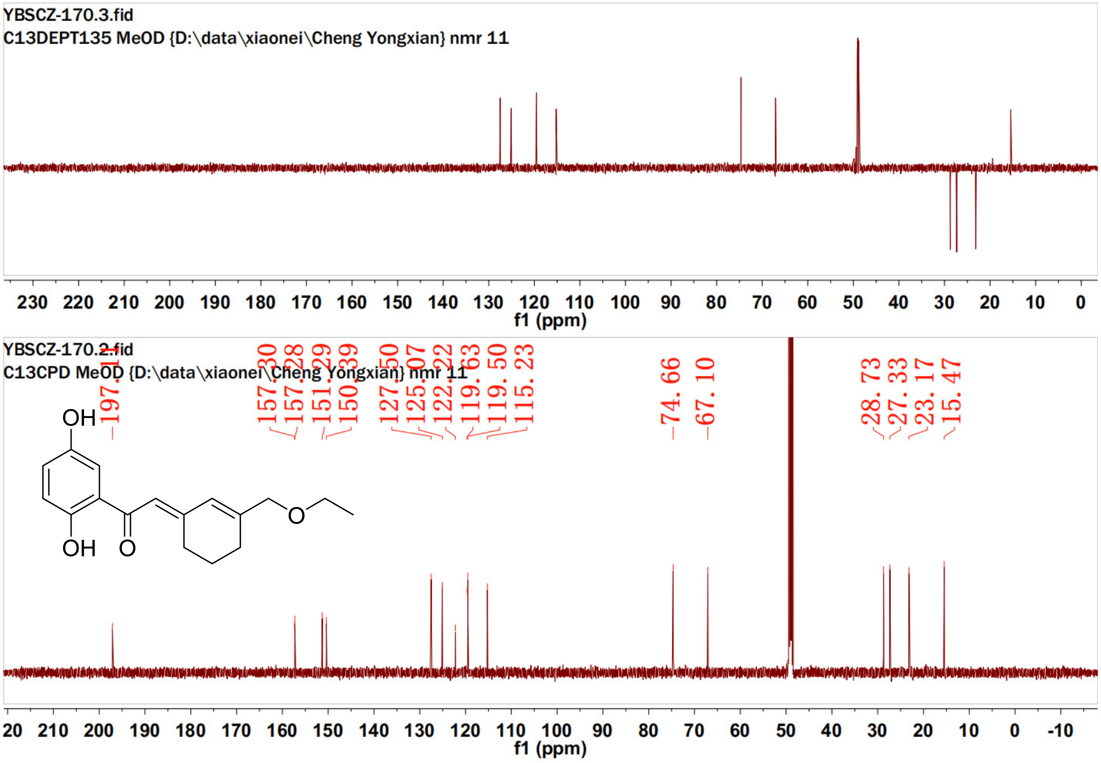

Figure S11. ^{13}C NMR and DEPT spectra of **2** in methanol-d_4.

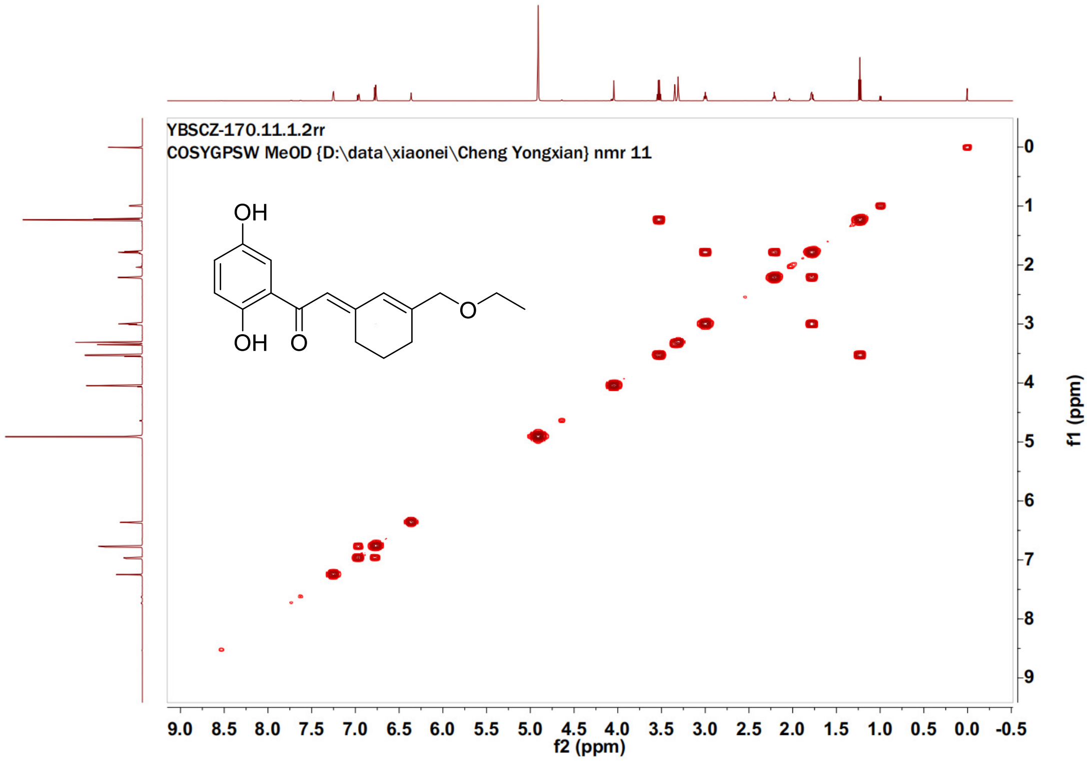

Figure S12. ^{1}H-^{1}H COSY spectrum of **2** in methanol-d_4.

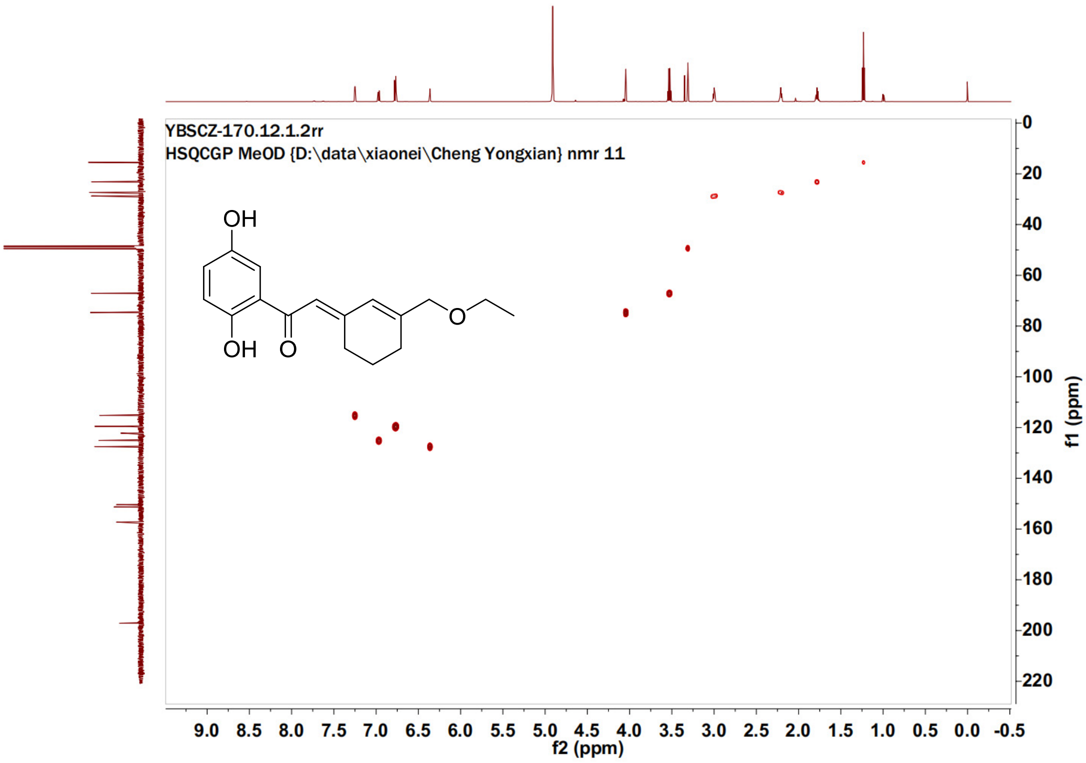

Figure S13. HSQC spectrum of **2** in methanol-d_4.

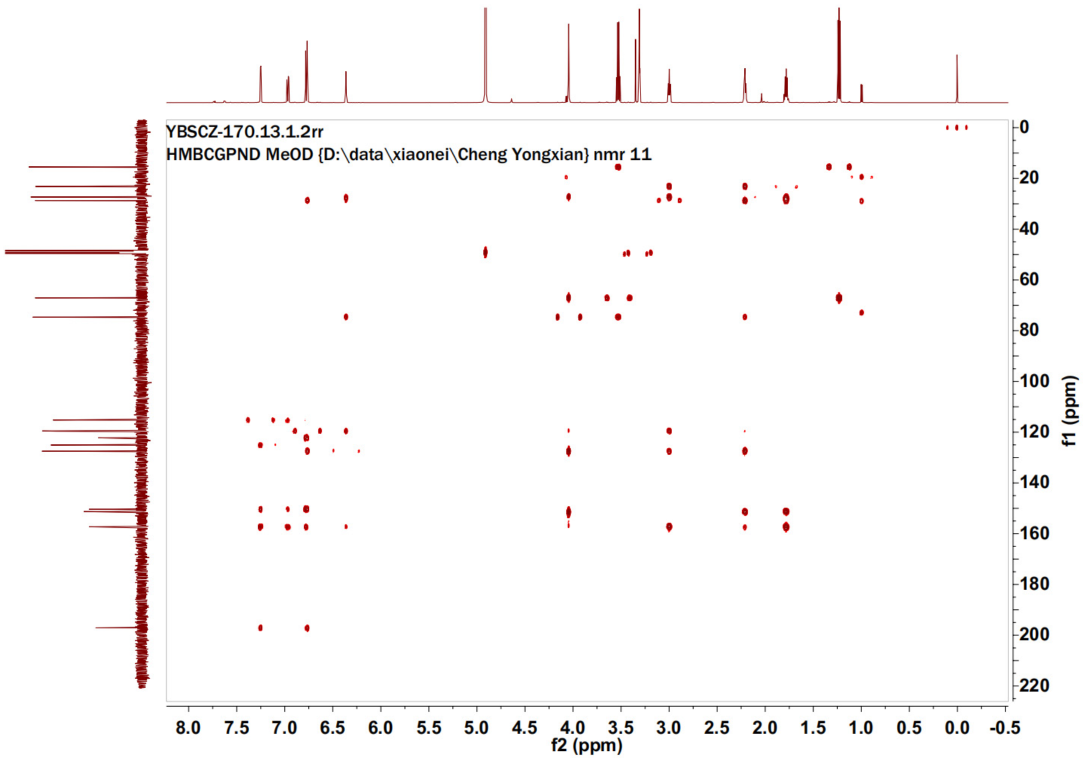

Figure S14. HMBC spectrum of **2** in methanol-d_4.

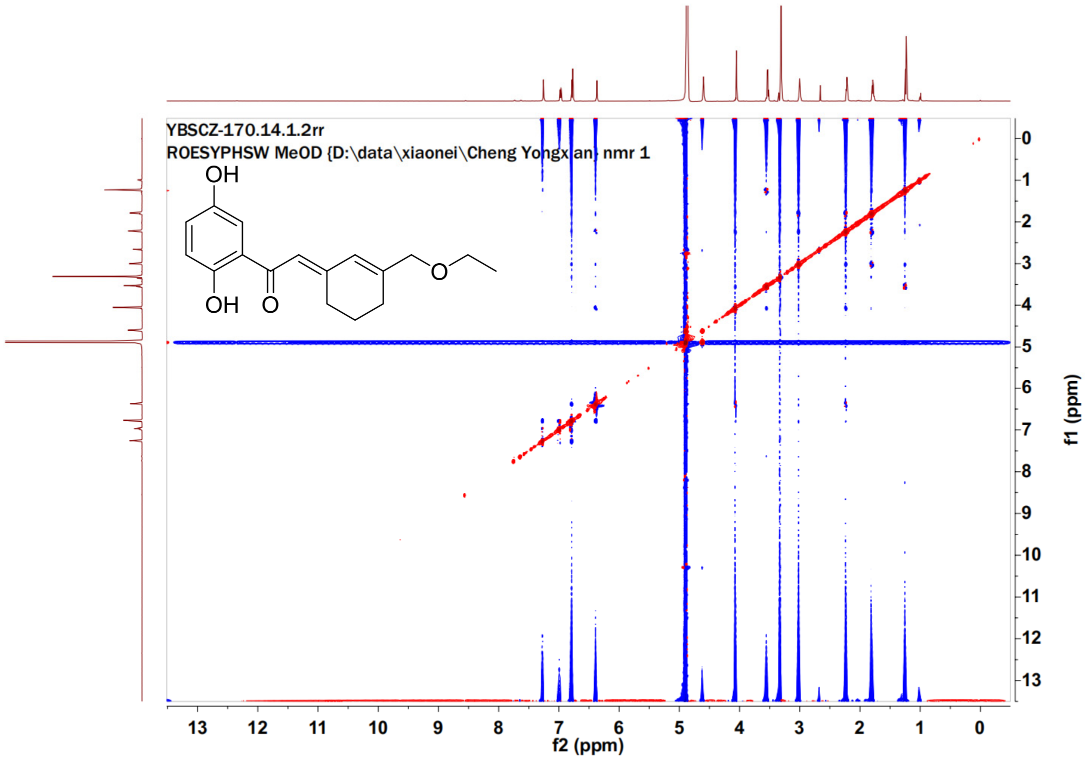

Figure S15. ROESY spectrum of **2** in methanol-*d*4.

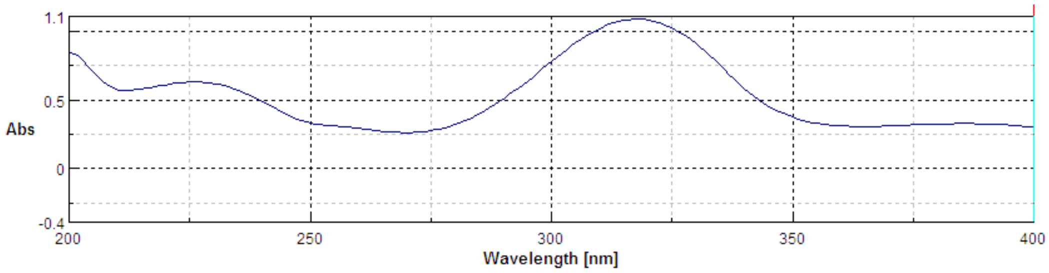

Figure S16. UV spectrum of **2**.

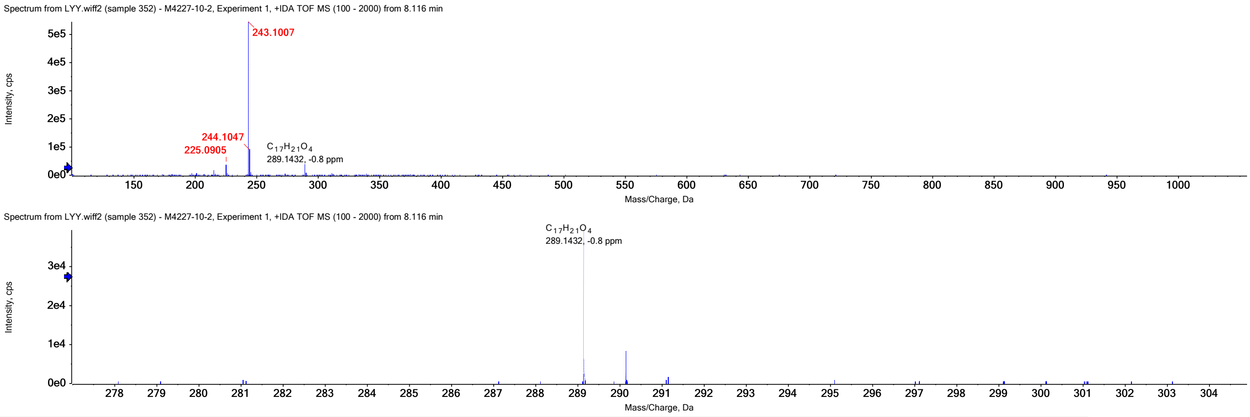

Hit	Formula	m/z	RDB	ppm	MS Rank	MSMS ppm	MSMS Rank	Found
1	C17H20O4	289.1434	8.0	-0.8	1			NA/NA

Figure S17. HRESIMS of **2**.

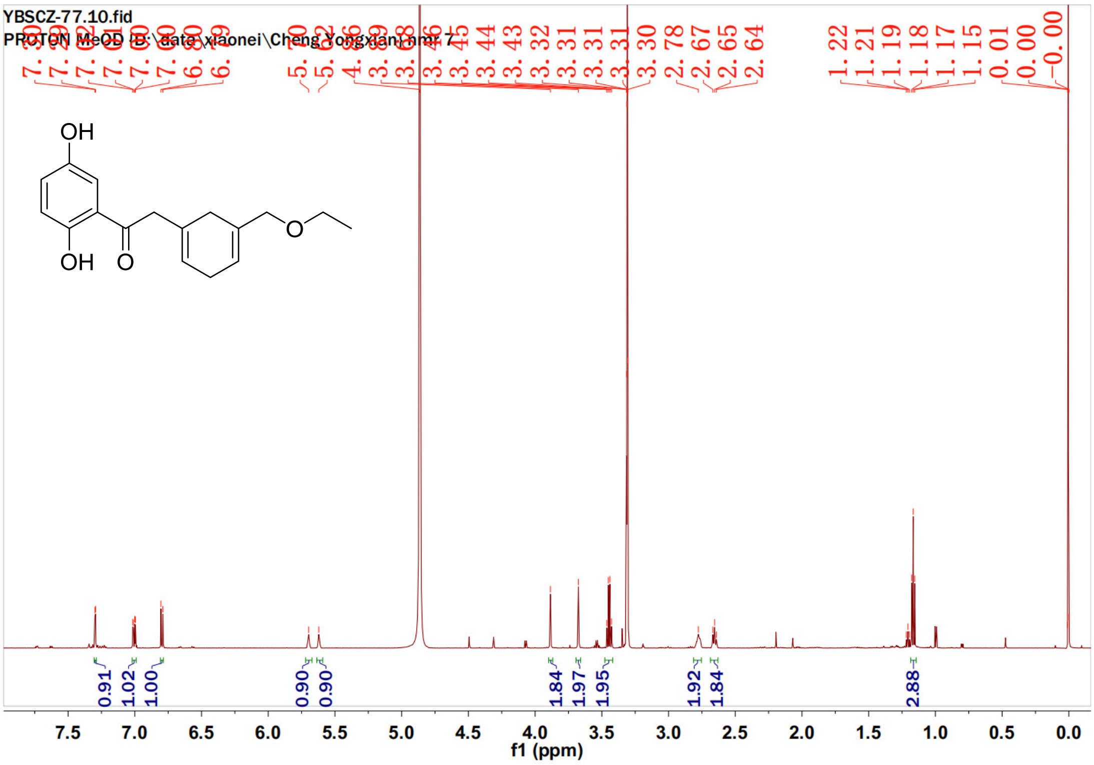

Figure S18. ^{1}H NMR spectrum of **3** in methanol-d_4.

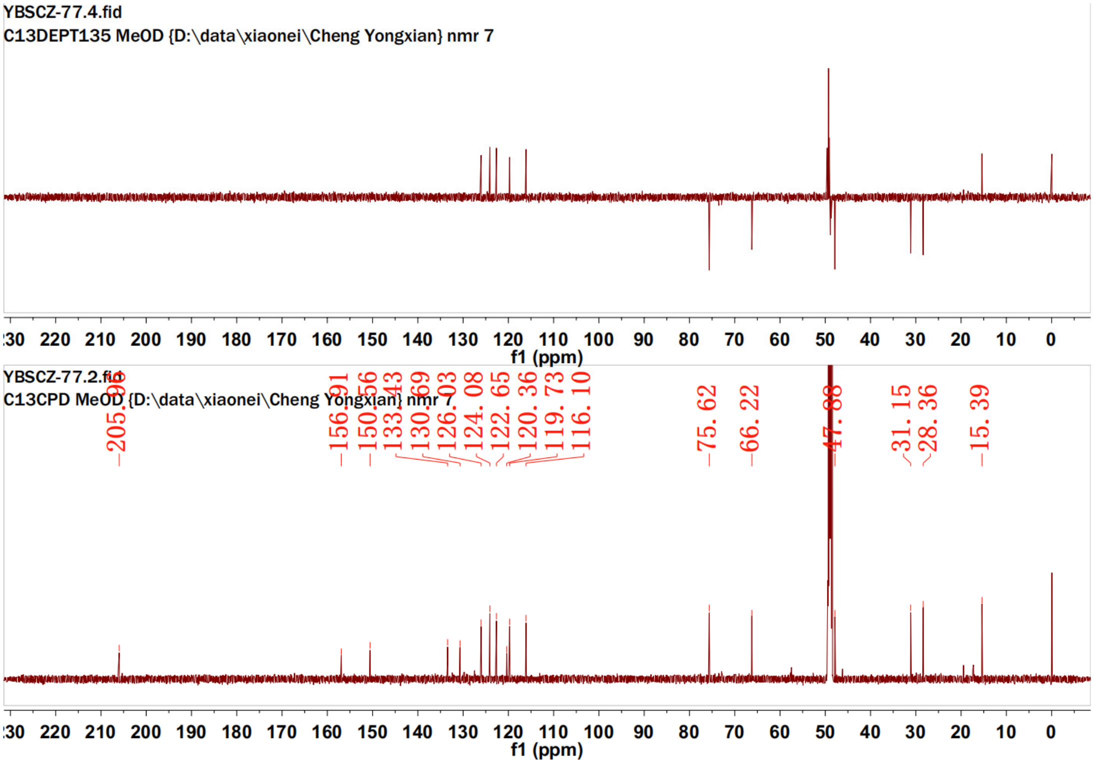

Figure S19. ^{13}C NMR and DEPT spectra of **3** in methanol-d_4.

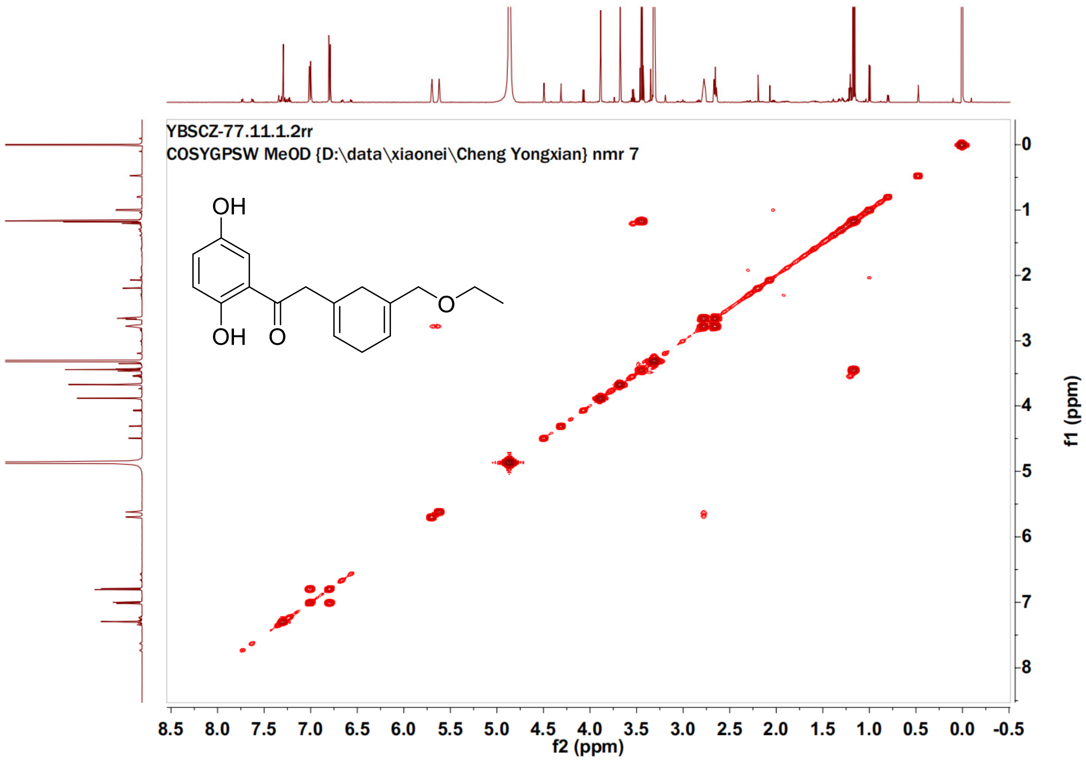

Figure S20. ^{1}H-^{1}H COSY spectrum of **3** in methanol-*d*4.

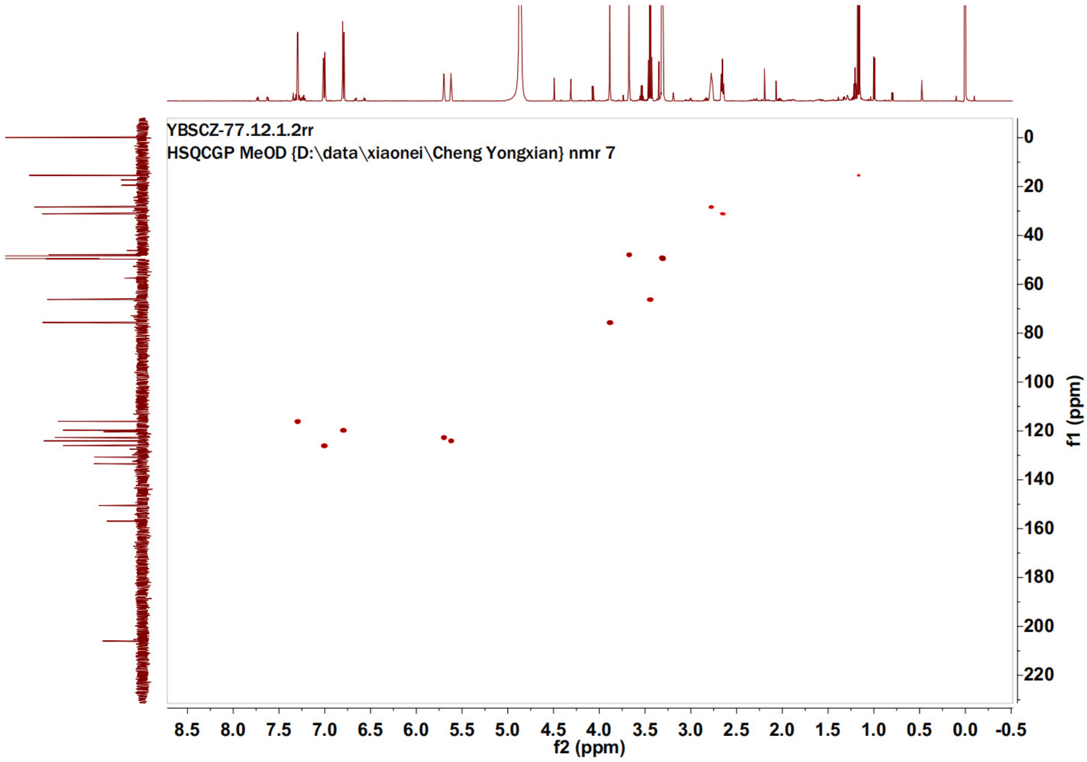

Figure S21. HSQC spectrum of **3** in methanol-*d*4.

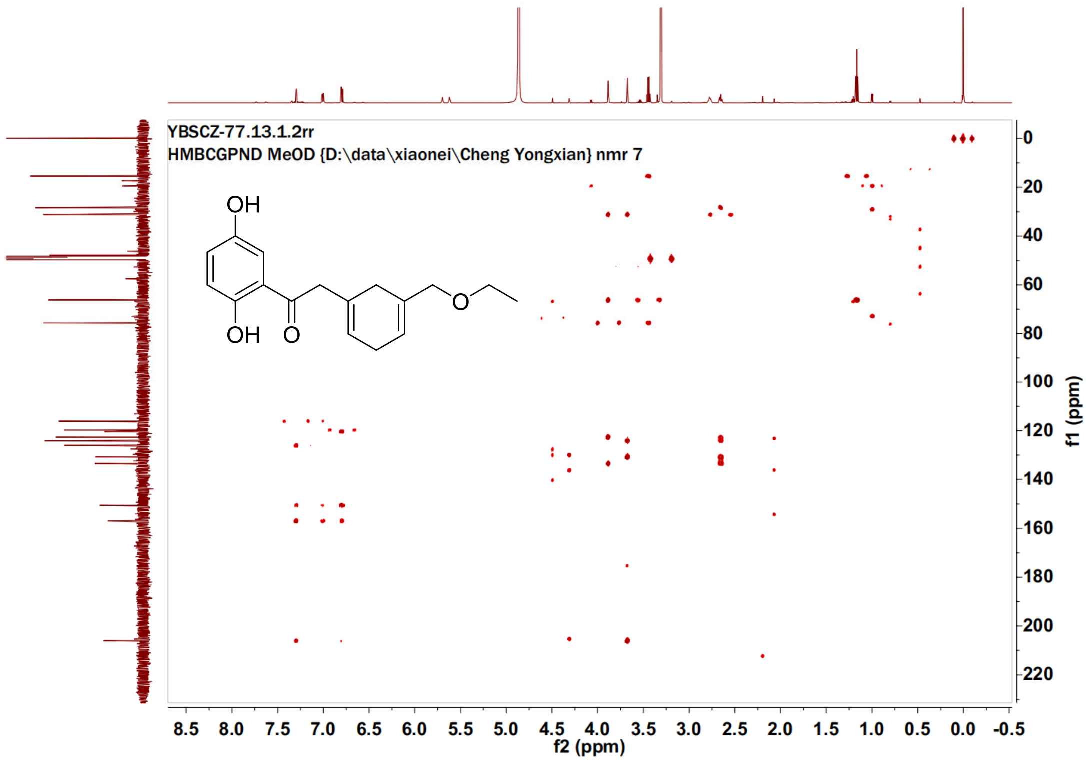

Figure S22. HMBC spectrum of **3** in methanol-d_4.

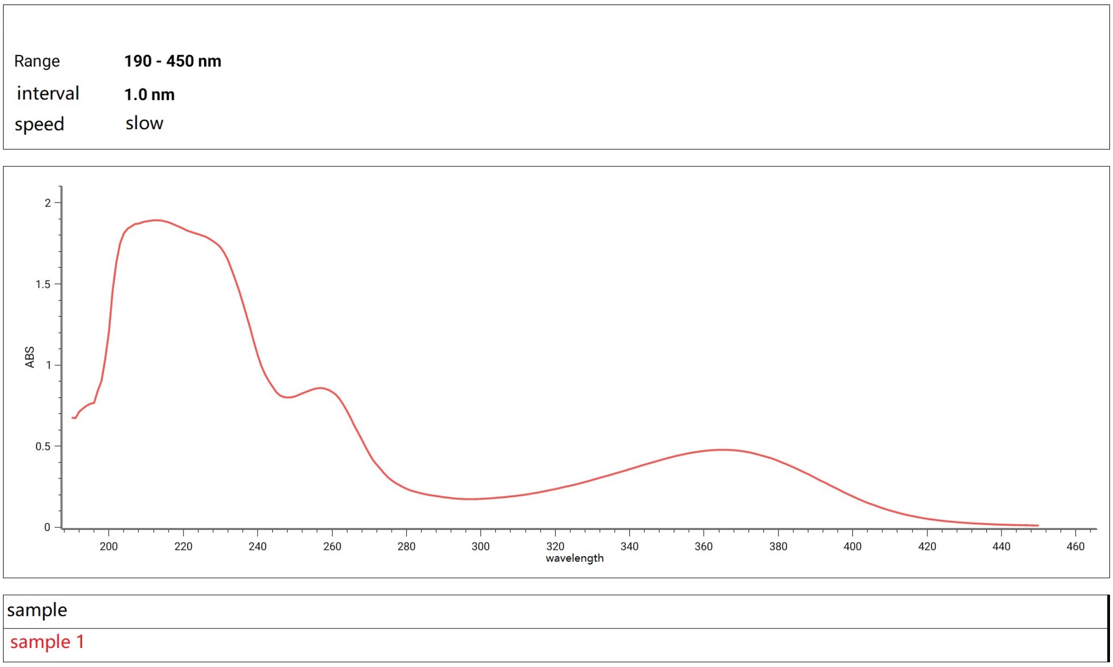

Figure S23. UV spectrum of **3**.

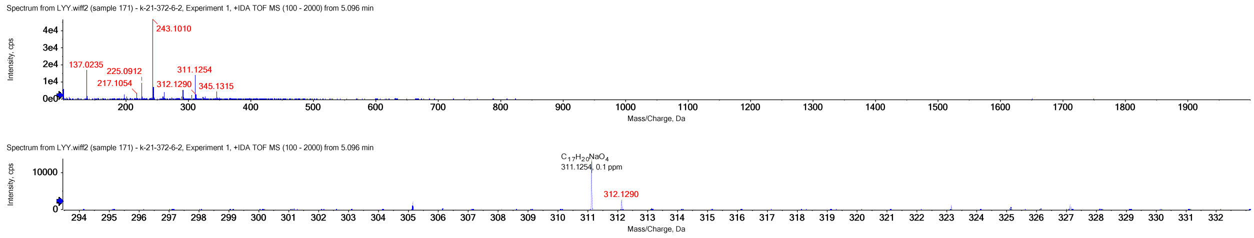

Hit	Formula	m/z	RDB	ppm	MS Rank	MSMS ppm	MSMS Rank	Found
1	C17H20O4	311.1254	8.0	0.1	1			NA/NA

Figure S24. HRESIMS of **3**.

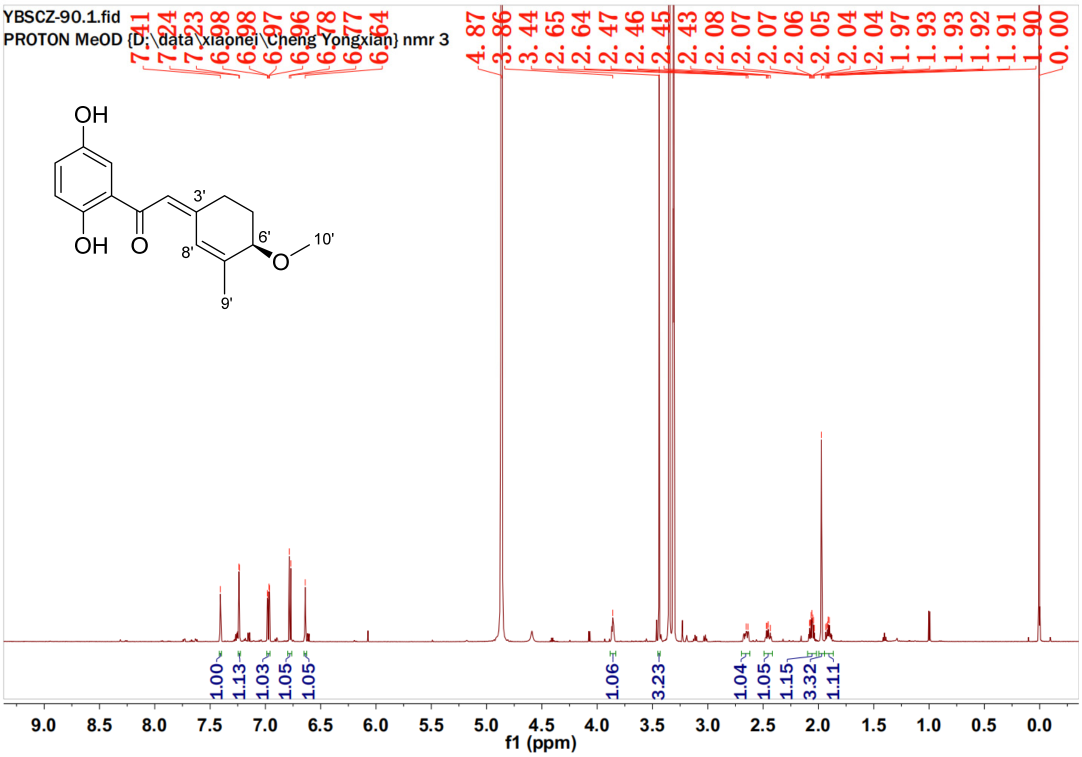

Figure S25. ^{1}H NMR spectrum of **4** in methanol-d_4.

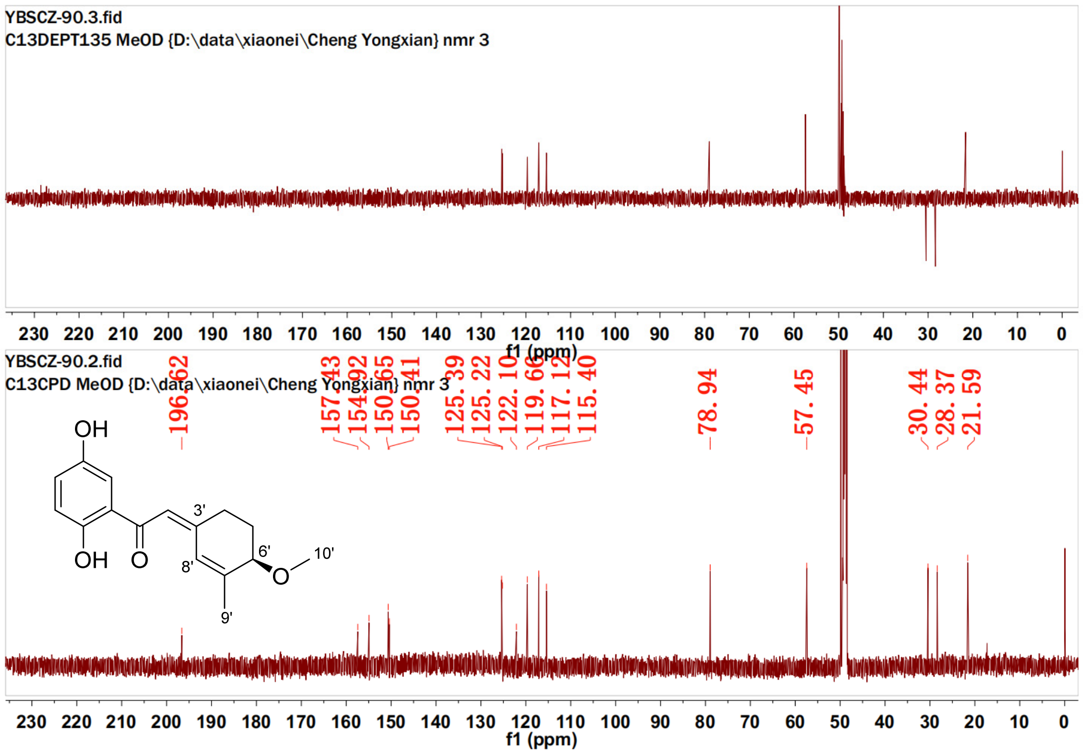

Figure S26. ^{13}C NMR and DEPT spectra of **4** in methanol-d_4.

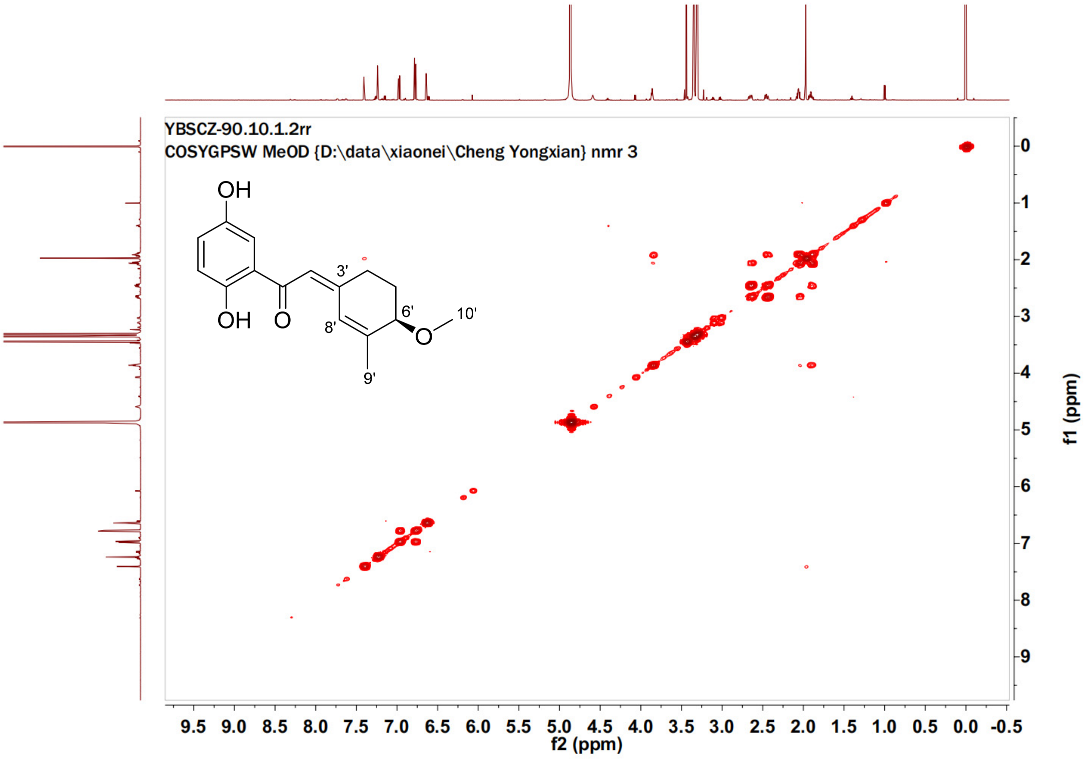

Figure S27. ^{1}H-^{1}H COSY spectrum of **4** in methanol-d_4.

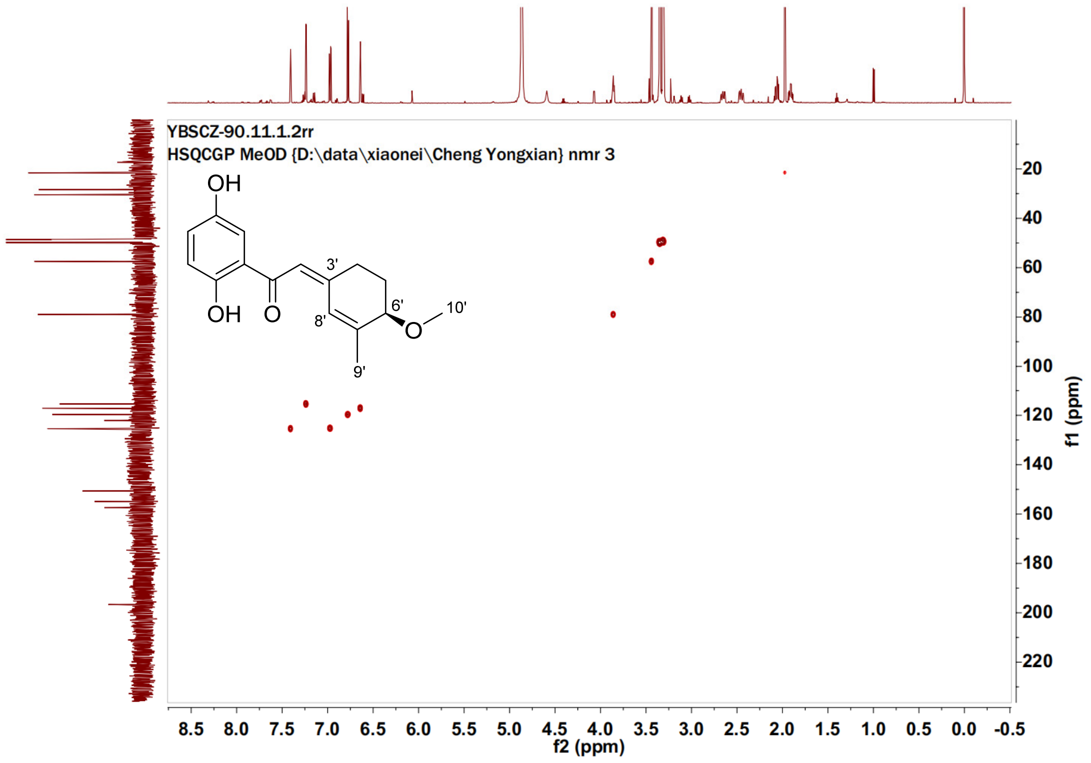

Figure S28. HSQC spectrum of **4** in methanol-d_4.

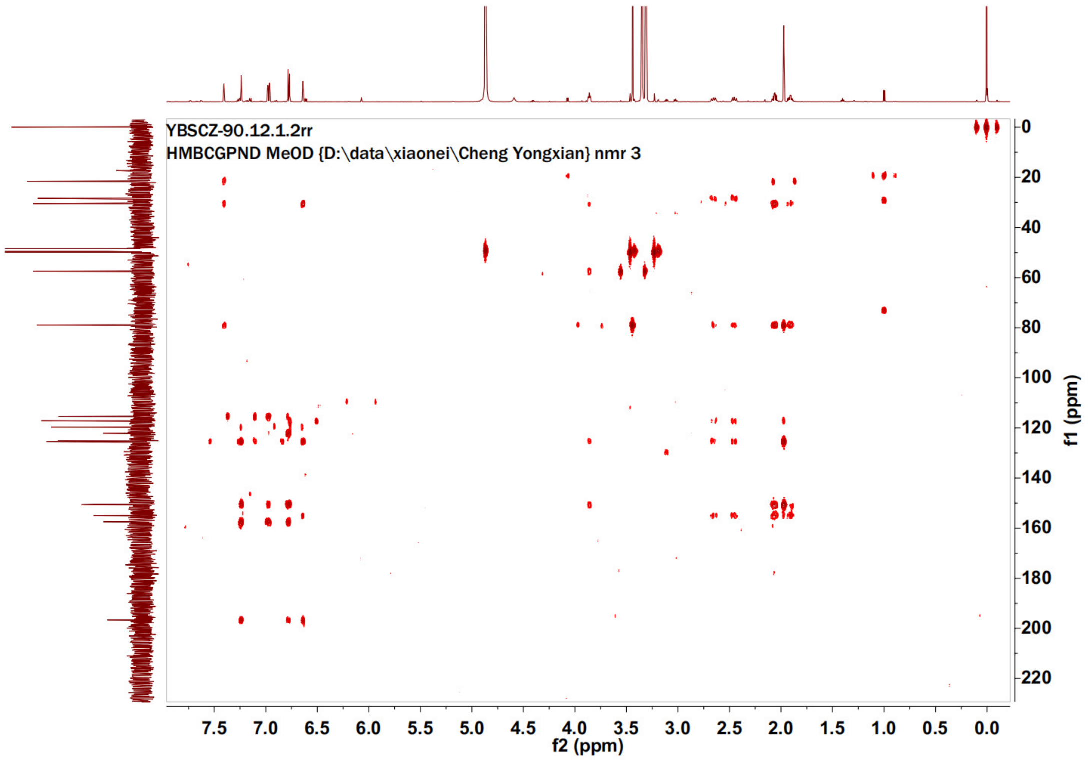

Figure S29. HMBC spectrum of **4** in methanol-d_4.

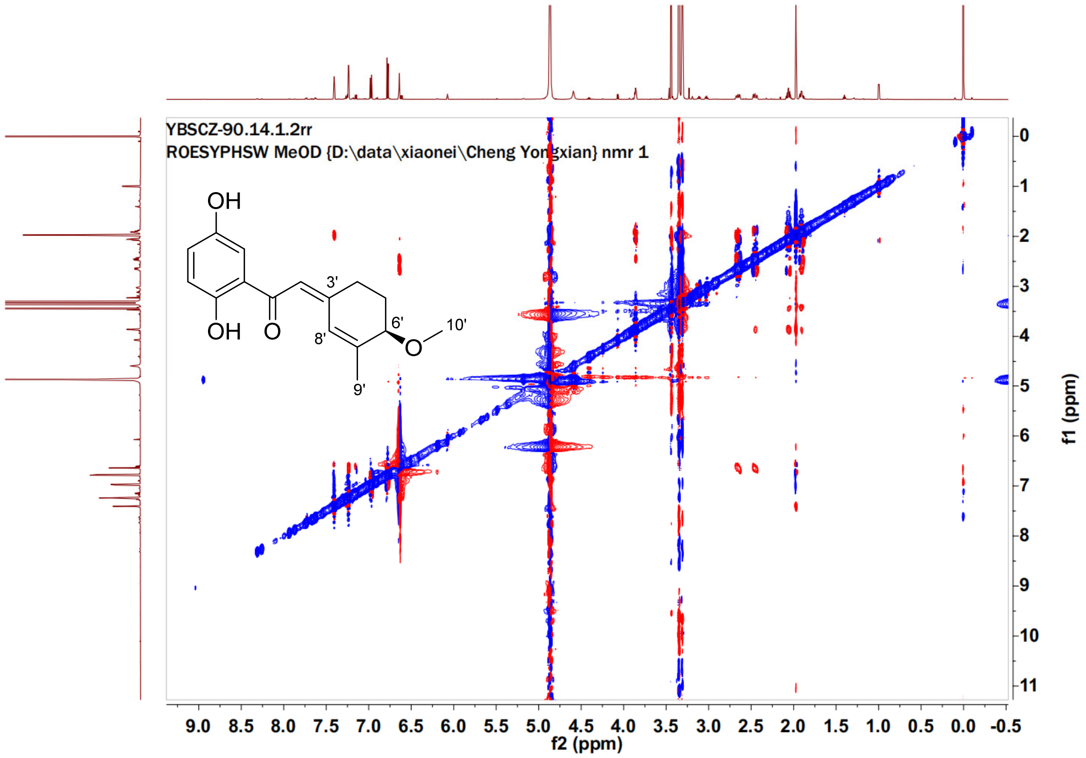

Figure S30. ROESY spectrum of **4** in methanol-d_4.

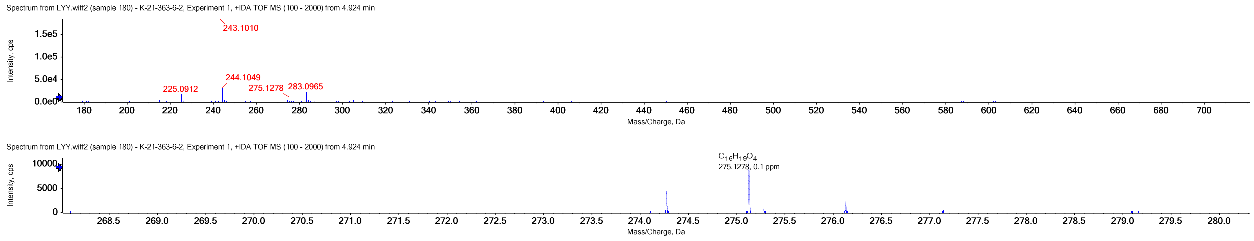

Hit	Formula	m/z	RDB	ppm	MS Rank	MSMS ppm	MSMS Rank	Found
1	C16H18O4	275.1278	8.0	0.1	1			NA/NA

Figure S31. HRESIMS of **4.**

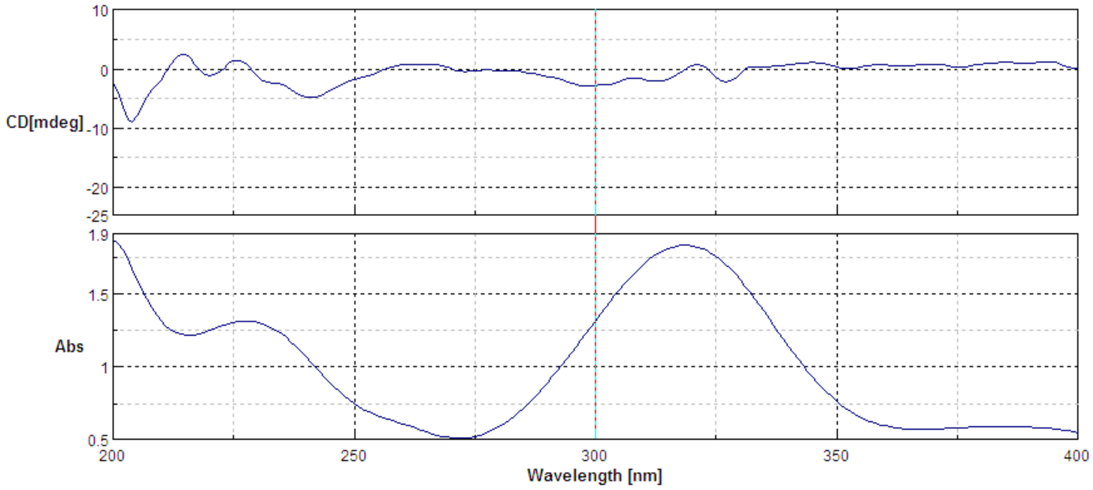

Figure S32. CD and UV spectra of (+)-**4**.

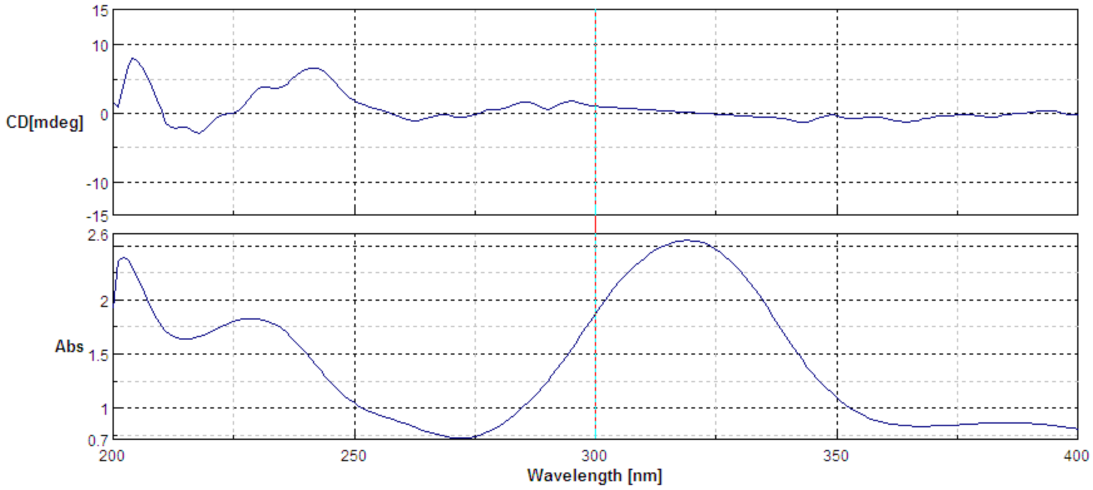

Figure S33. CD and UV spectra of (–)-**4**.

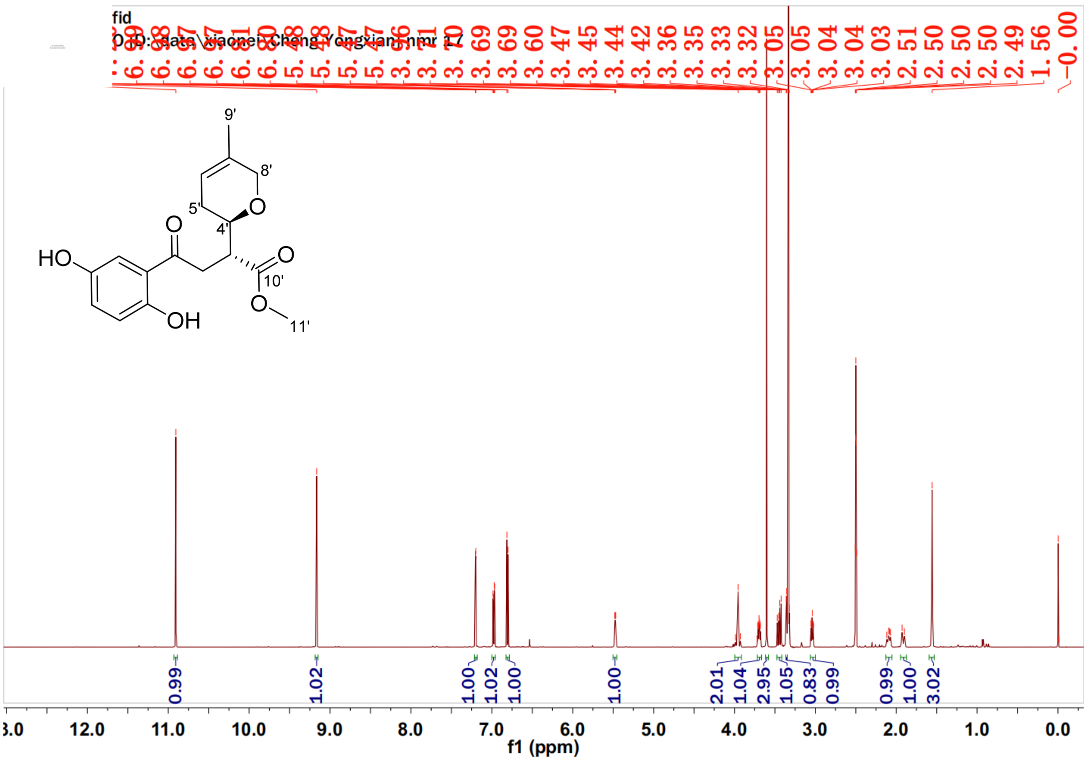

Figure S34. ^{1}H NMR spectrum of **5** in DMSO-d_6.

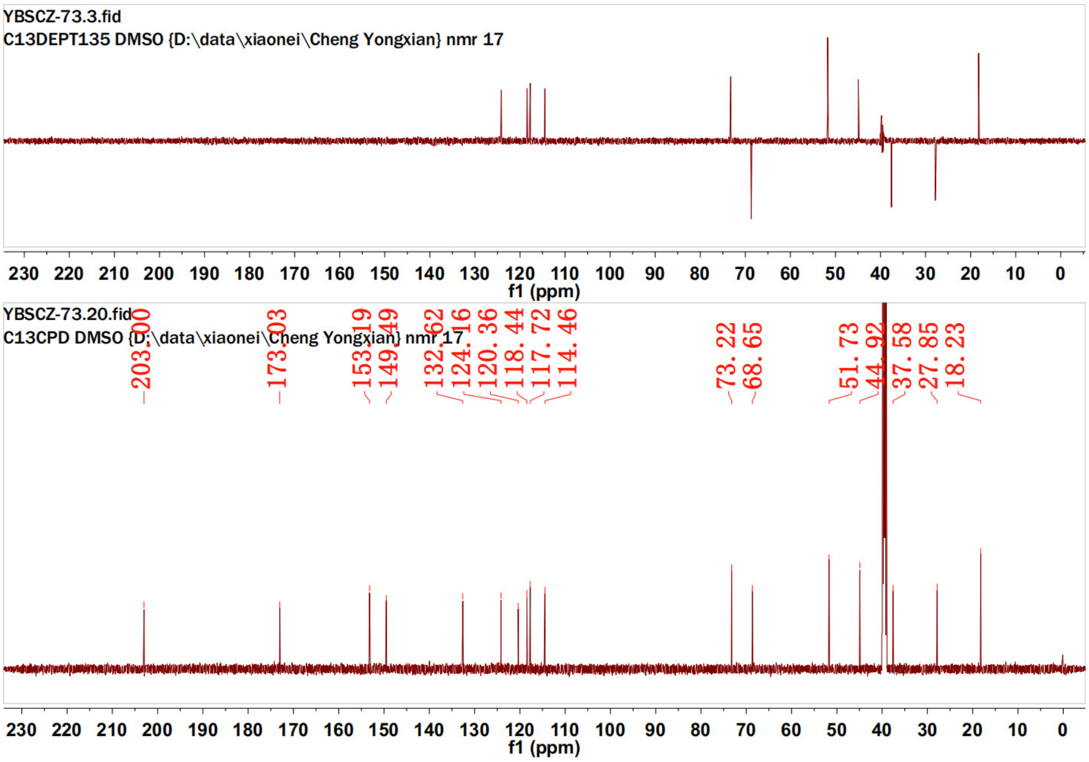

Figure S35. ^{13}C NMR and DEPT spectra of **5** in DMSO-d_6.

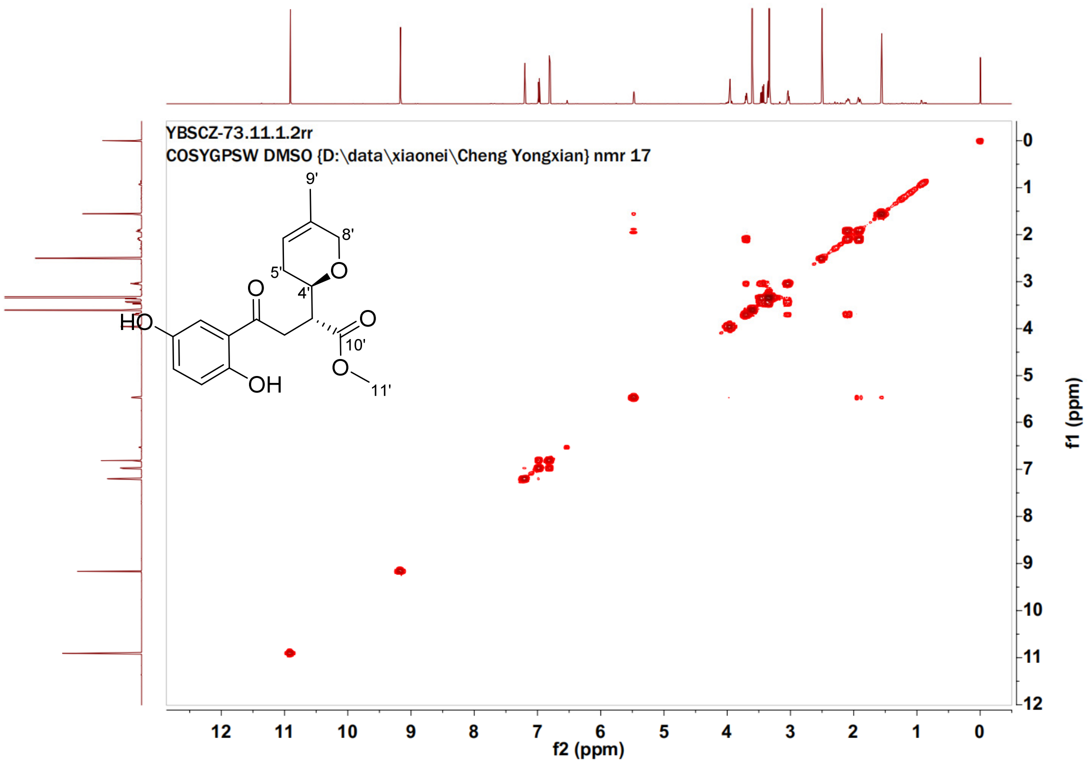

Figure S36. ¹H-¹H COSY spectra of **5** in DMSO-d_6.

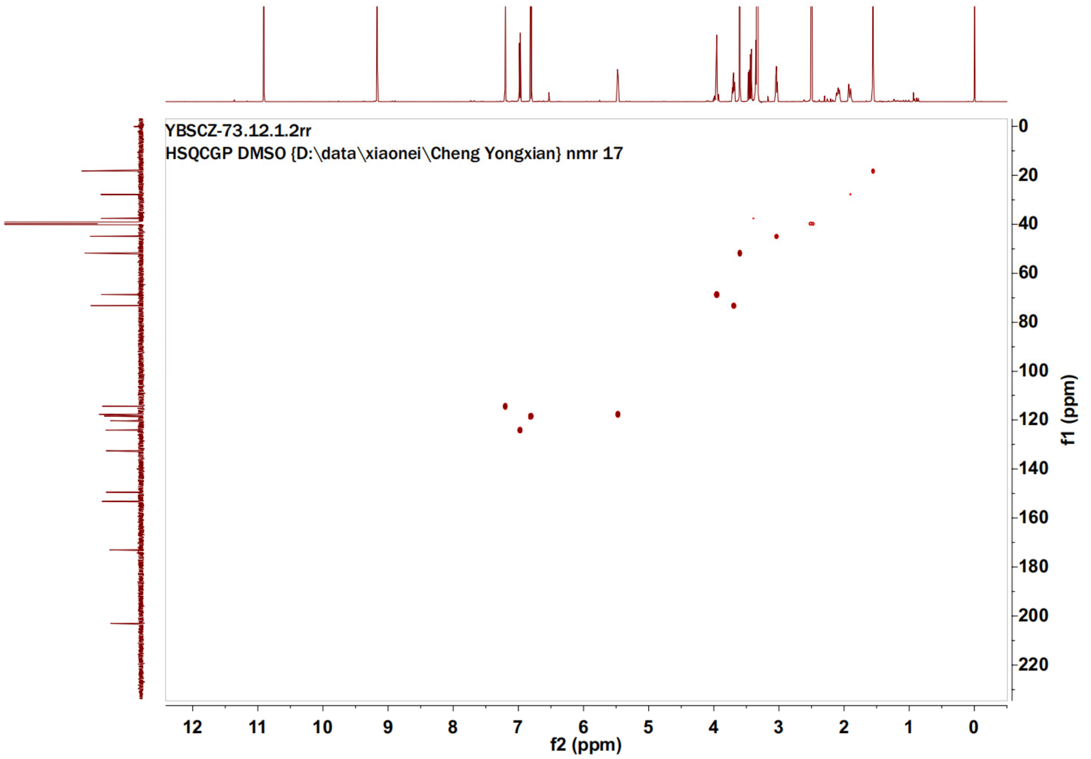

Figure S37. HSQC spectrum of **5** in DMSO-d_6.

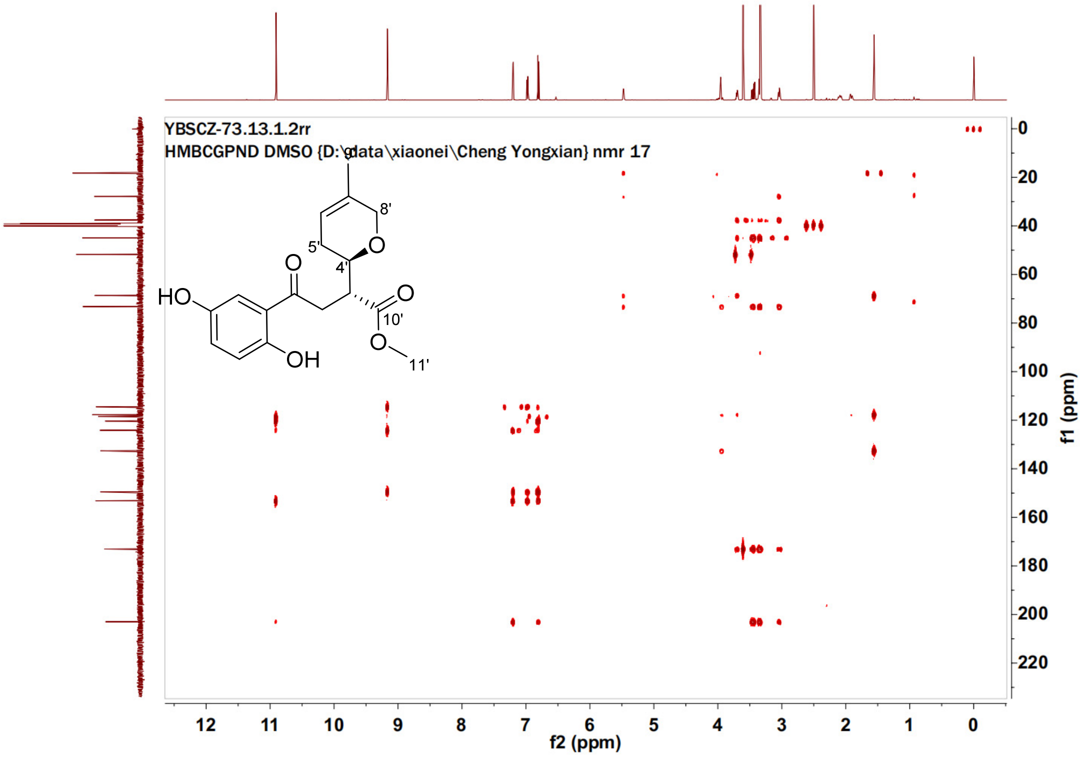

Figure S38. HMBC spectrum of **5** in DMSO-d_6.

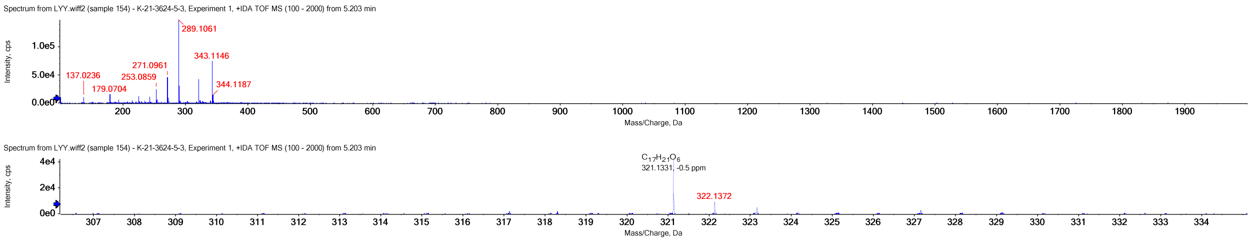

Hit	Formula	m/z	RDB	ppm	MS Rank	MSMS ppm	MSMS Rank	Found
1	C17H20O6	321.1333	8.0	-0.5	1			NA/NA

Figure S39. HRESIMS of **5**.

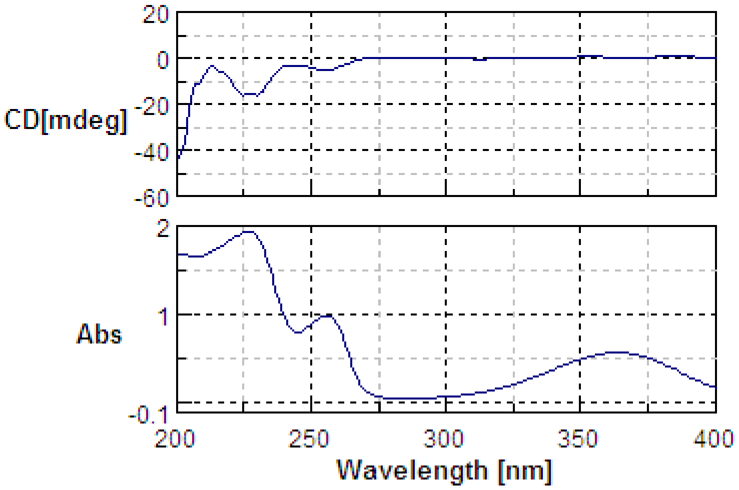

Figure S40. CD and UV spectra of (–)-**5**.

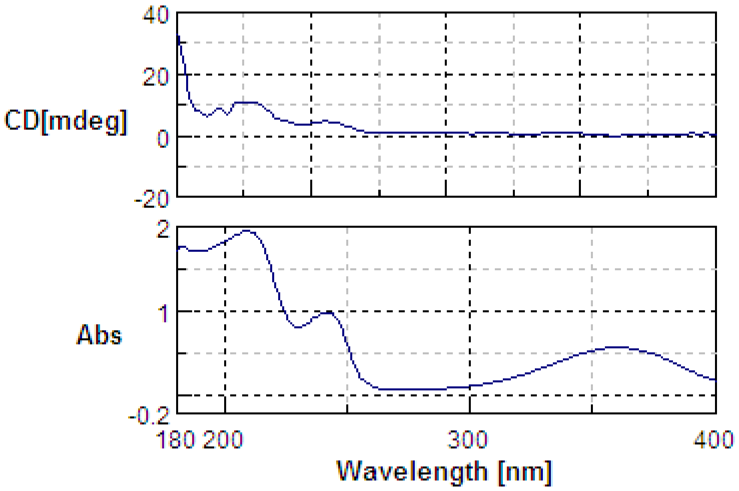

Figure S41. CD and UV spectra of (+)-**5**.

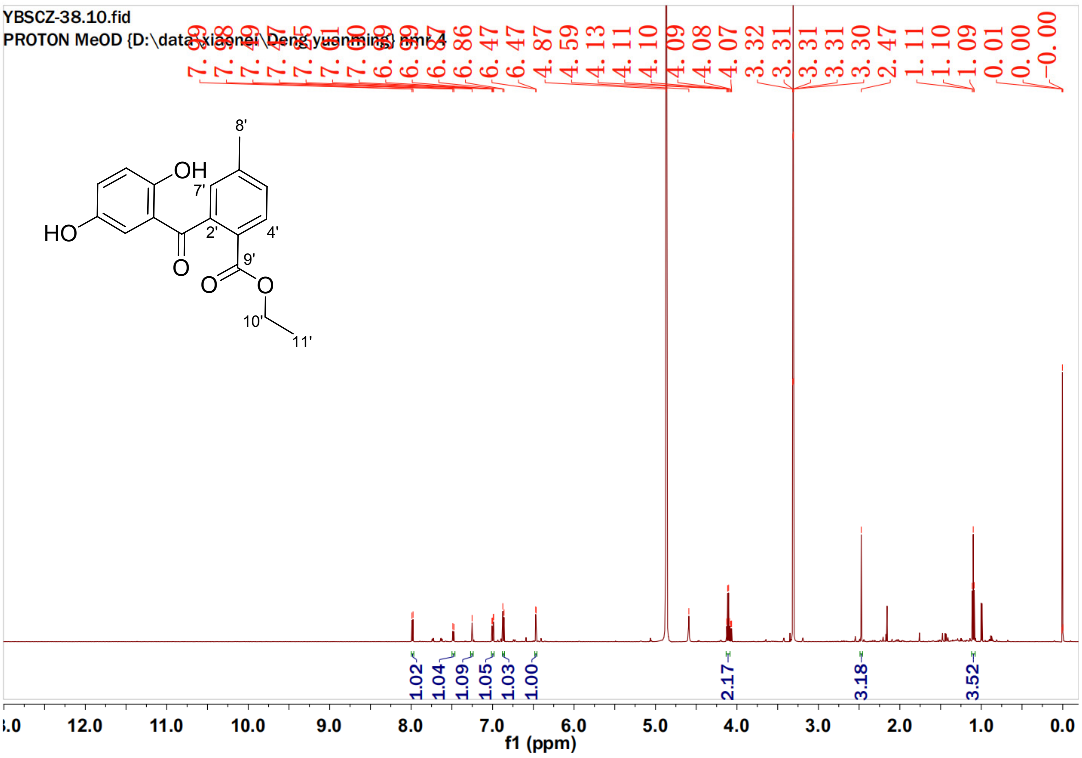

Figure S42. ^{1}H NMR spectrum of **6** in methanol-d_4.

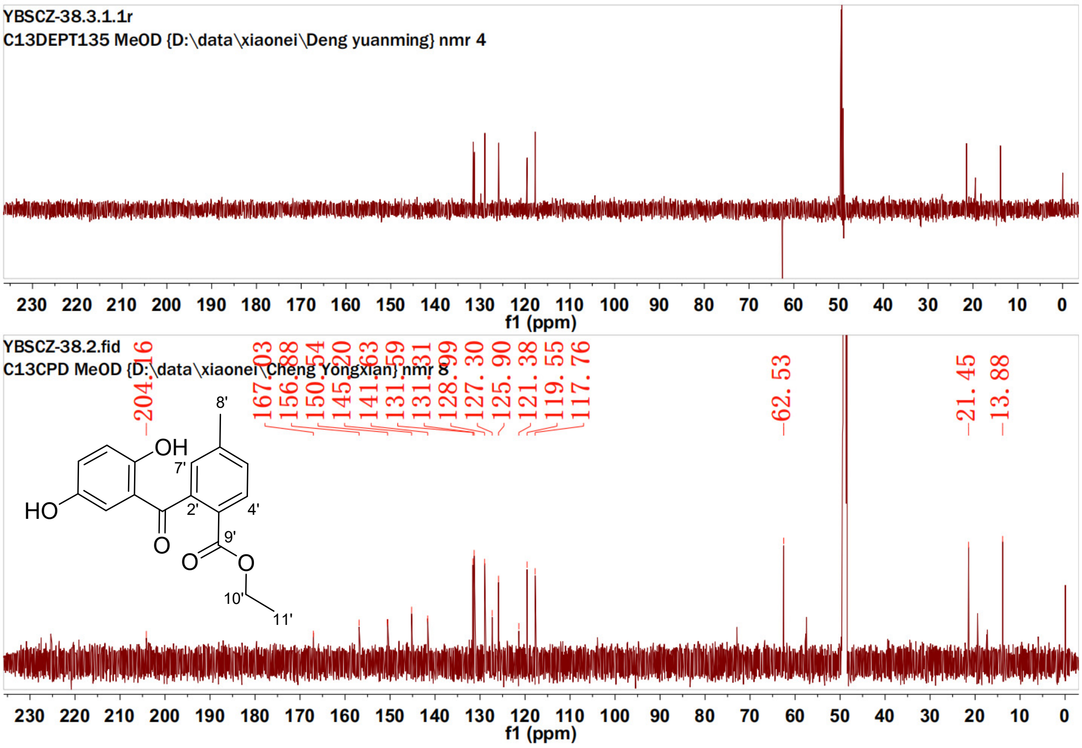

Figure S43. ^{13}C NMR and DEPT spectra of **6** in methanol-d_4.

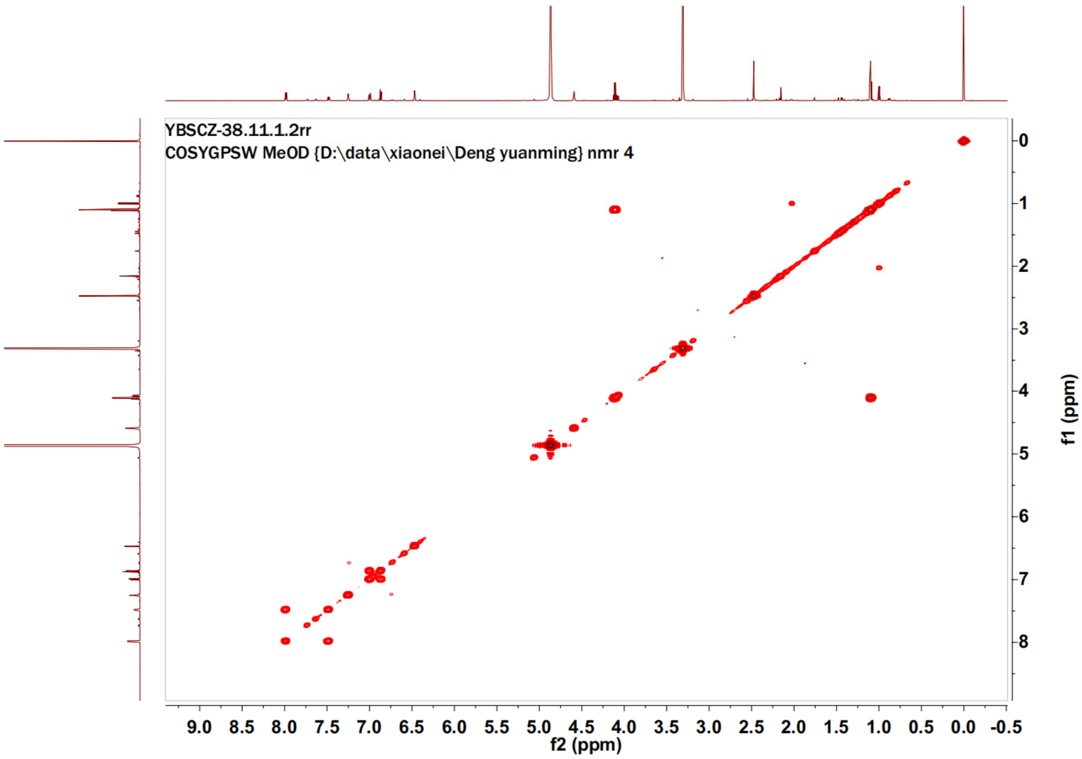

Figure S44. ^{1}H-^{1}H COSY spectrum of **6** in methanol-d_4.

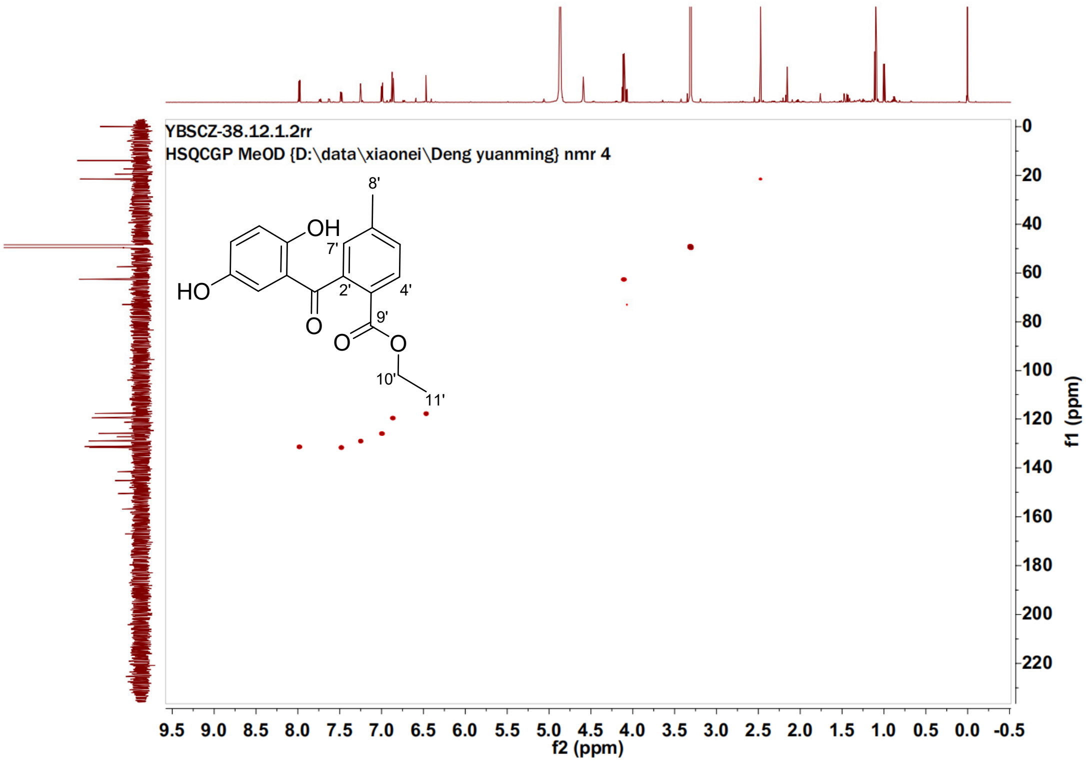

Figure S45. HSQC spectrum of **6** in methanol-*d*4.

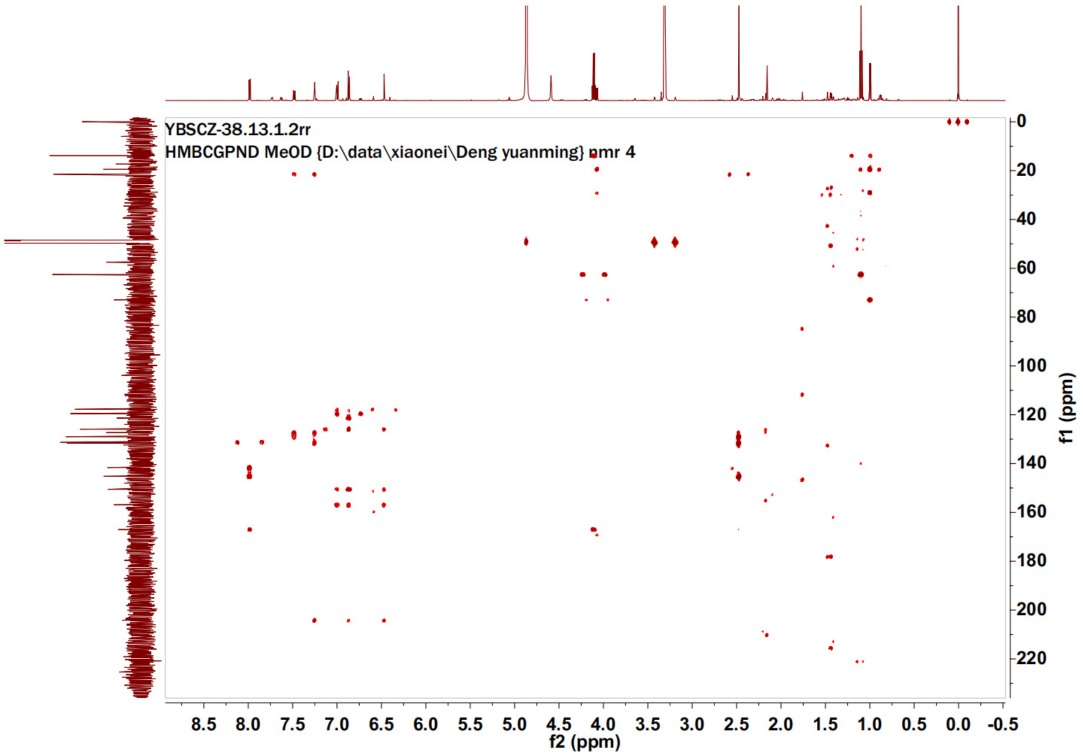

Figure S46. HMBC spectrum of **6** in methanol-*d*4.

Range	**190 - 450 nm**
interval	**1.0 nm**
speed	slow

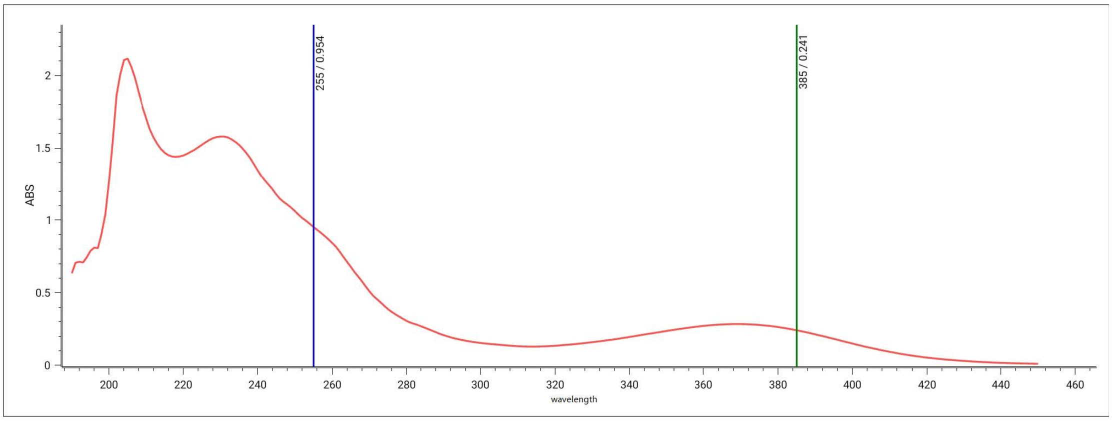

sample	ABS(255)	ABS(385)
sample 1	0.954	0.241

Figure S47. UV spectrum of **6**.

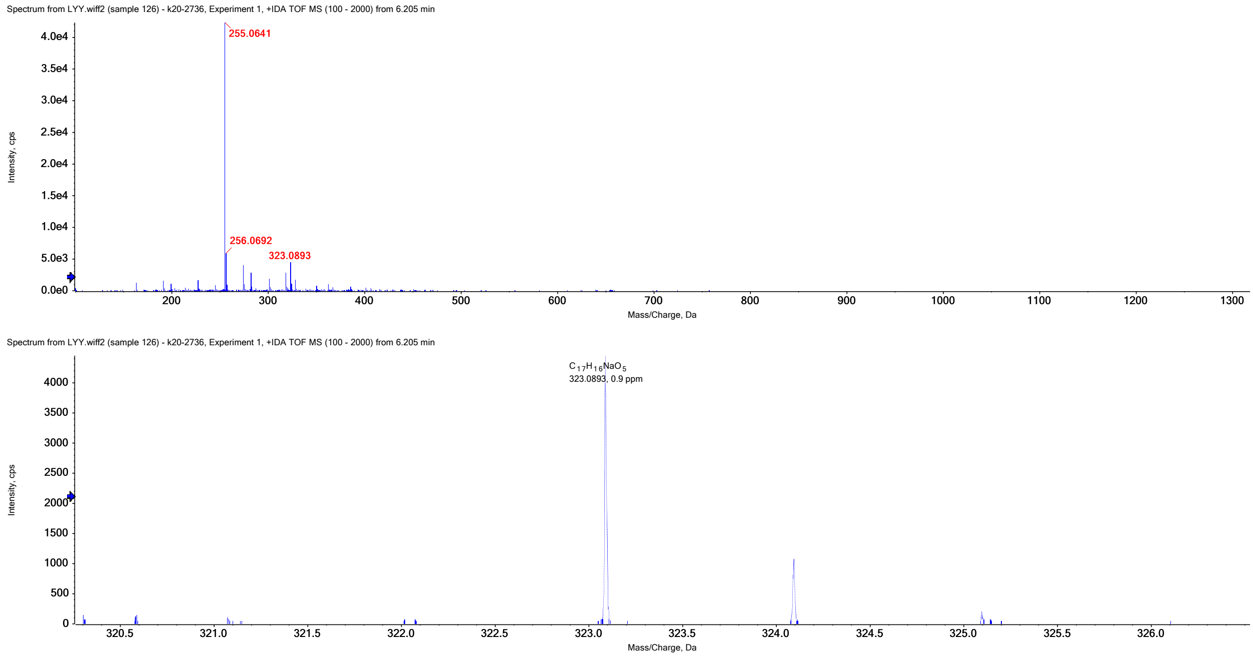

Hit	Formula	m/z	RDB	ppm	MS Rank	MSMS ppm	MSMS Rank	Found
1	C17H16O5	323.0890	10.0	0.9	1			NA/NA

Figure S48. HRESIMS of **6.**

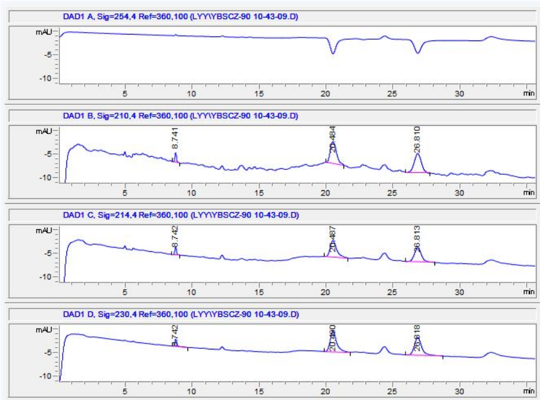

Figure S49. Chiral HPLC separation of racemic **4** (*n*-hexane/ethanol, 95:5; flow rate: 3.0 mL/min; wavelength: 254 nm, 210 nm, 214 nm, 230 nm and 260 nm).

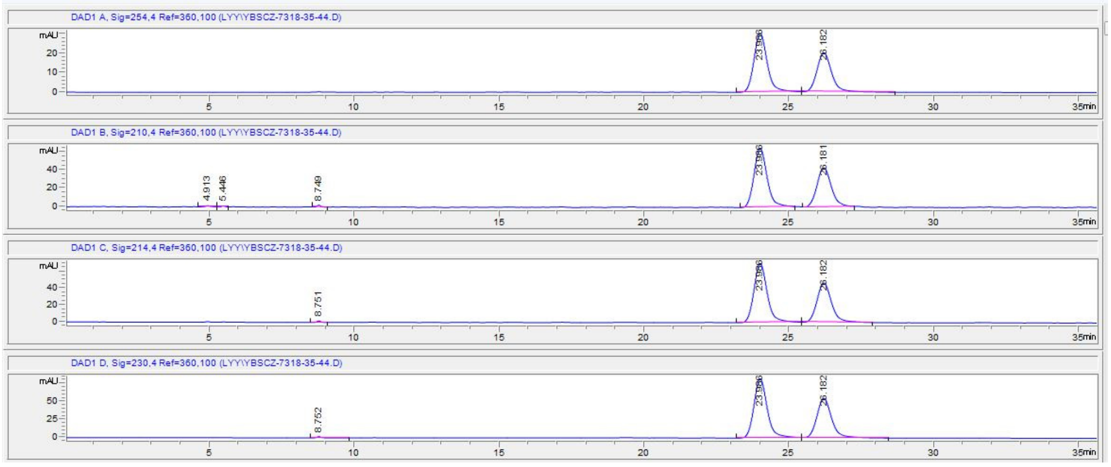

Figure S50. Chiral HPLC separation of racemic **5** (*n*-hexane/ethanol, 95:5; flow rate: 3.0 mL/min; wavelength: 254 nm, 210 nm, 214 nm, 230 nm and 260 nm).

ECD calculations for compounds 4 and 5.

Conformation search using molecular mechanics calculations was performed in CONFLEX version 7.0 with MMFF force field with an energy window for acceptable conformers (ewindow) of 5 kcal/mol above the ground state, a maximum number of conformations per molecule (maxconfs) of 100, and an RMSD cutoff (rmsd) of 0.5Å. Then the predominant conformers were optimized at B3LYP/6-311g(d,p) level in Gaussian 09 (Frisch et al. 2009) [1]. The optimized conformation geometries and thermodynamic parameters of all selected conformations were provided. The optimized conformers of **4** and **5** were used for the ECD calculation, which were performed with Gaussian 09 [40] (B3LYP/6-311g(d,p)). The solvent effects were taken into account by the polarizable-conductor calculation model (PCM, methanol as the solvent). Percentages for each conformation are shown in Table S1.

Selected conformations of 4

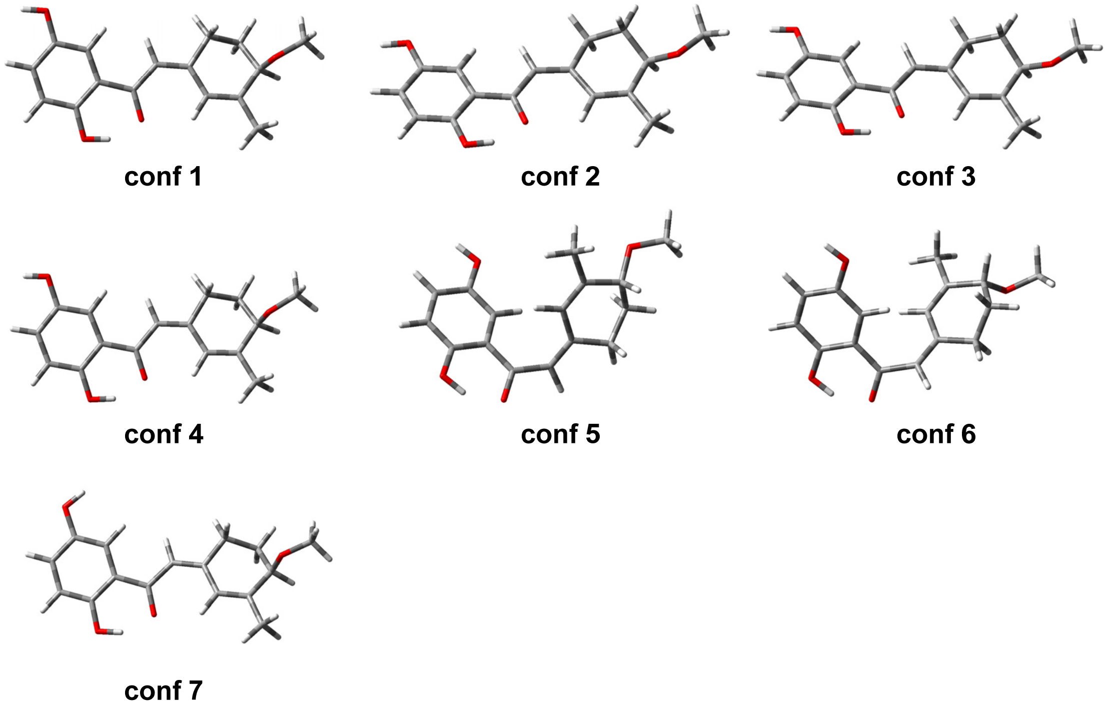

Figure S51. The lowest energy conformers of **4**.

Selected conformations of 5

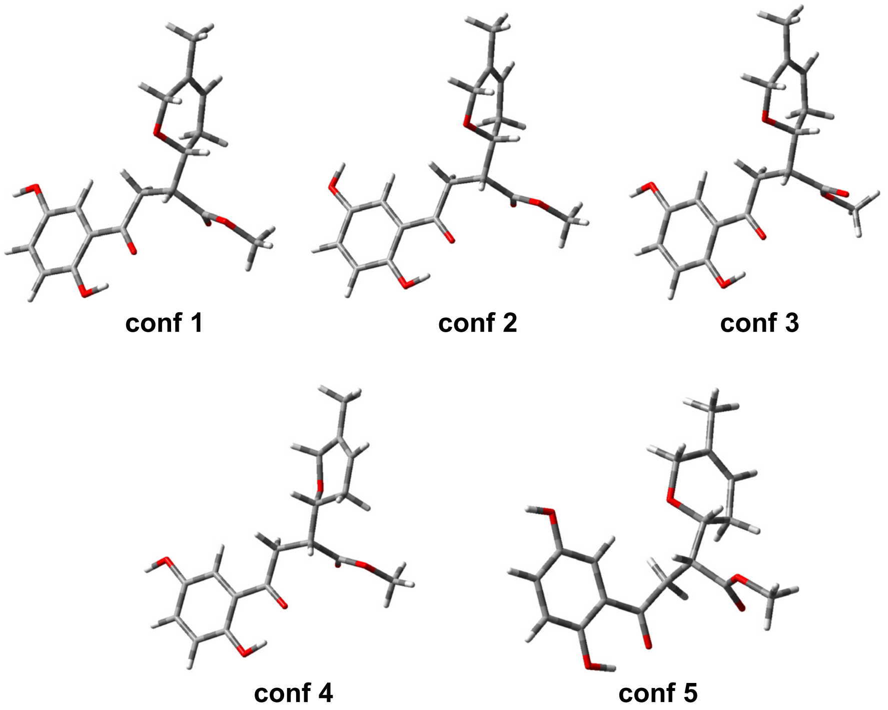

Figure S52. The lowest energy conformers of **5**.

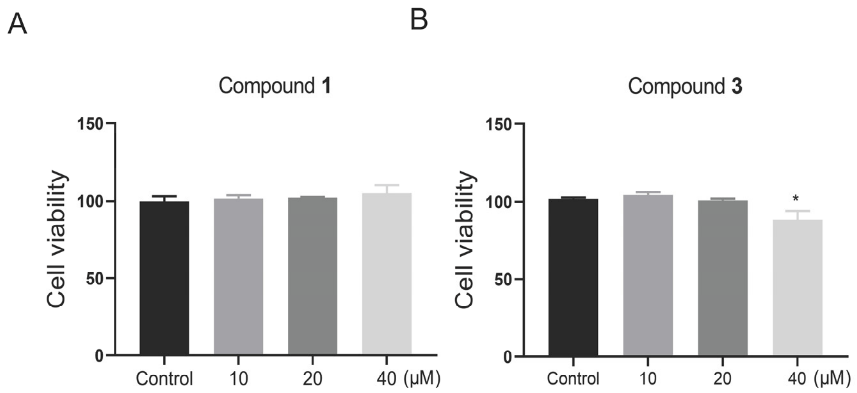

Figure S53 The cytotoxic effects of compounds **1** and **3** were detected in RAW264.7 cells with different concentrations (10, 20 and 40 μM). Three independent experiments were performed. ANOVA. *P < 0.05 vs control group.

Table S1. Extracted heats and weighting factors of the optimized conformers of **4** and **5** at B3LYP/6-311g(d,p) level

		B3LYP/6-311g(d,p)	
	Conformer	Extracted heats	Boltzmann-calculated contribution (%)
**4**	1	-921.4064	18.06%
	2	-921.40667	24.02%
	3	-921.40667	24.04%
	4	-921.4064	18.08%
	5	-921.3994	0.01%
	6	-921.39924	0.01%
	7	-921.40627	15.79%
**5**	1	-1111.21195	35.67%
	2	-1111.21167	26.51%
	3	-1111.21035	6.52%
	4	-1111.21181	30.69%
	5	-1111.20812	0.62%

Table S2. The Cartesian coordinates of the lowest energy conformers for **4** and **5**

The Cartesian coordinates of the lowest energy conformer for **4**							
**conf 1**	X axis(Å)	Y axis(Å)	Z axis(Å)	**conf 2**	X axis(Å)	Y axis(Å)	Z axis(Å)
C	-4.844	0.7657	0.007	C	4.8261	-0.6082	-0.3289
C	-5.1487	-0.565	-0.2751	C	5.012	0.7626	-0.501
C	-4.1153	-1.4887	-0.3931	C	3.9198	1.6164	-0.3869
C	-2.785	-1.1112	-0.2237	C	2.6482	1.1285	-0.0935
C	-2.4717	0.2199	0.0805	C	2.4566	-0.2457	0.1008
C	-3.5155	1.1559	0.1741	C	3.5567	-1.1083	-0.0391
O	-3.3088	2.486	0.4304	O	3.4648	-2.4687	0.0966
O	-4.3652	-2.7979	-0.6816	O	4.0519	2.9625	-0.5599
C	-1.0662	0.6683	0.2796	C	1.118	-0.811	0.4249
C	-0.0413	-0.3487	0.6512	C	0.0897	0.0941	1.0124
C	1.2791	-0.2802	0.3944	C	-1.2444	0.004	0.8491

C	2.2006	-1.3857	0.8708	C	-2.1556	0.9933	1.5465
C	3.5186	-0.819	1.3946	C	-3.2994	1.4319	0.6414
C	4.2239	0.0154	0.3179	C	-4.1159	0.2275	0.1571
C	3.2685	0.9113	-0.4368	C	-3.2312	-0.9193	-0.2845
C	1.9303	0.7784	-0.361	C	-1.9222	-0.987	0.0277
C	3.9033	1.9855	-1.2739	C	-3.895	-2.0106	-1.0789
O	4.8394	-0.8472	-0.6446	O	-4.9257	0.6305	-0.9525
C	6.1873	-1.1605	-0.3229	C	-6.159	1.2152	-0.5589
O	-0.8104	1.8677	0.2017	O	0.9275	-2.0149	0.2705
H	-5.6375	1.504	0.0913	H	5.6657	-1.2919	-0.4255
H	-6.1865	-0.8553	-0.4056	H	6.0045	1.1386	-0.7288
H	-2.0159	-1.8665	-0.353	H	1.8258	1.8354	-0.0413
H	-2.3389	2.6523	0.3922	H	2.5104	-2.7002	0.1677
H	-5.3229	-2.9162	-0.7993	H	4.9763	3.1604	-0.7868
H	-0.4251	-1.2036	1.2003	H	0.4916	0.8862	1.638
H	1.7296	-1.975	1.6671	H	-2.5627	0.5245	2.452
H	2.3953	-2.0745	0.0386	H	-1.6009	1.882	1.8712
H	3.3175	-0.1899	2.2717	H	-3.9494	2.1309	1.1808
H	4.1557	-1.6466	1.7255	H	-2.9078	1.9928	-0.2185
H	4.9833	0.6422	0.8045	H	-4.7536	-0.1465	0.9698
H	1.2996	1.4802	-0.9021	H	-1.3396	-1.8255	-0.3466
H	4.5129	2.6447	-0.6473	H	-4.766	-2.3956	-0.5386
H	4.5461	1.5429	-2.0416	H	-4.2244	-1.6354	-2.0529
H	3.1589	2.6067	-1.7829	H	-3.2221	-2.8554	-1.2613
H	6.2546	-1.6469	0.6543	H	-5.9934	2.1215	0.03
H	6.5697	-1.8488	-1.0814	H	-6.7121	1.4868	-1.4621
H	6.8015	-0.2549	-0.3361	H	-6.7582	0.4985	0.0109
**conf 3**	X axis(Å)	Y axis(Å)	Z axis(Å)	**conf 4**	X axis(Å)	Y axis(Å)	Z axis(Å)
C	-4.8275	0.9462	-0.2885	C	4.8466	-0.7624	-0.0318
C	-5.2544	-0.3347	0.0582	C	5.1307	0.5744	0.2426
C	-4.3074	-1.3133	0.3417	C	4.0836	1.4838	0.3497
C	-2.9429	-1.0404	0.2738	C	2.7598	1.0859	0.1762
C	-2.5054	0.2384	-0.095	C	2.467	-0.2515	-0.121
C	-3.464	1.2321	-0.3549	C	3.5246	-1.1729	-0.2027
O	-3.1369	2.5212	-0.6843	O	3.3384	-2.5077	-0.4501
O	-4.6786	-2.5758	0.6994	O	4.313	2.7983	0.6312
C	-1.0582	0.5749	-0.19	C	1.0685	-0.7214	-0.3233
C	-0.0832	-0.5333	-0.4068	C	0.034	0.2796	-0.7139
C	1.2239	-0.5303	-0.0801	C	-1.2886	0.2137	-0.467
C	2.0813	-1.7378	-0.4	C	-2.2029	1.3192	-0.9597
C	3.4711	-1.3259	-0.8684	C	-3.2436	1.6891	0.0949
C	4.1742	-0.4569	0.1817	C	-4.08	0.4666	0.4928
C	3.2522	0.5983	0.7562	C	-3.2372	-0.7756	0.6708

C	1.914	0.5511	0.6056	C	-1.9583	-0.8566	0.2552
C	3.9049	1.7083	1.5335	C	-3.9112	-1.9281	1.3605
O	5.2937	0.1971	-0.4245	O	-5.0436	0.1796	-0.5257
C	6.4622	-0.6097	-0.4565	C	-6.2899	0.8249	-0.3057
O	-0.7248	1.7566	-0.1536	O	0.8309	-1.9229	-0.2291
H	-5.553	1.7273	-0.5016	H	5.6509	-1.4898	-0.1074
H	-6.3185	-0.5432	0.1091	H	6.1637	0.8803	0.3763
H	-2.2472	-1.833	0.531	H	1.9794	1.831	0.2963
H	-2.1653	2.6262	-0.5625	H	2.3709	-2.6879	-0.4159
H	-5.6494	-2.6212	0.7268	H	5.2693	2.9324	0.7439
H	-0.494	-1.4084	-0.9021	H	0.4184	1.1333	-1.265
H	2.1668	-2.3624	0.4989	H	-1.6305	2.2184	-1.219
H	1.6164	-2.3586	-1.1756	H	-2.704	0.9871	-1.8782
H	4.0704	-2.2204	-1.0749	H	-2.7328	2.0873	0.9815
H	3.4047	-0.7851	-1.8226	H	-3.8827	2.4867	-0.2995
H	4.5156	-1.0813	1.0187	H	-4.5873	0.6856	1.4421
H	1.3117	1.3561	1.0211	H	-1.4023	-1.7703	0.449
H	4.5381	1.2943	2.3251	H	-4.2375	-1.6325	2.3629
H	4.5241	2.3285	0.8778	H	-4.7874	-2.2554	0.7917
H	3.1697	2.3649	2.0109	H	-3.2457	-2.7911	1.4694
H	6.2991	-1.5135	-1.0498	H	-6.1668	1.9105	-0.2583
H	7.2642	-0.0312	-0.923	H	-6.9521	0.5859	-1.1422
H	6.773	-0.8749	0.5586	H	-6.7506	0.4574	0.6164
**conf 5**	X axis(Å)	Y axis(Å)	Z axis(Å)	**conf 6**	X axis(Å)	Y axis(Å)	Z axis(Å)
C	-3.7708	1.1351	0.781	C	3.9112	0.7886	0.8845
C	-3.2107	2.2328	0.1292	C	3.5322	1.945	0.2041
C	-2.1242	2.0398	-0.718	C	2.4688	1.8904	-0.6914
C	-1.5804	0.7732	-0.9183	C	1.7714	0.7053	-0.9124
C	-2.1273	-0.332	-0.2543	C	2.1342	-0.4561	-0.2183
C	-3.2383	-0.1385	0.5831	C	3.2228	-0.4045	0.6674
O	-3.8628	-1.1608	1.2483	O	3.675	-1.4947	1.3633
O	-1.5541	3.0826	-1.3866	O	2.0719	2.9943	-1.3869
C	-1.5799	-1.7031	-0.4333	C	1.4162	-1.7432	-0.4164
C	-0.2363	-1.9098	-1.025	C	0.0725	-1.7735	-1.0431
C	0.929	-1.477	-0.5086	C	-1.0314	-1.1512	-0.5892
C	2.2312	-1.7687	-1.2215	C	-2.3396	-1.2714	-1.3423
C	3.1545	-0.5573	-1.1828	C	-3.0514	0.0787	-1.4061
C	3.4411	-0.1222	0.2608	C	-3.3139	0.6301	0.0022
C	2.1969	-0.141	1.1261	C	-2.14	0.4292	0.9354
C	1.0583	-0.7517	0.7456	C	-1.097	-0.3675	0.6346
C	2.3025	0.5395	2.4634	C	-2.2004	1.1855	2.2326
O	3.9663	1.2091	0.2553	O	-4.4324	-0.0437	0.5886
C	5.3639	1.2574	0.0053	C	-5.6675	0.6115	0.3366

O	-2.258	-2.679	-0.1154	O	1.9557	-2.7982	-0.086
H	-4.6263	1.266	1.4387	H	4.7464	0.8104	1.58
H	-3.637	3.2183	0.2877	H	4.0783	2.8665	0.38
H	-0.736	0.6747	-1.5938	H	0.9555	0.7105	-1.6285
H	-3.4793	-2.0087	0.9275	H	3.2004	-2.2879	1.0244
H	-2.0009	3.9053	-1.1258	H	2.6453	3.7404	-1.1433
H	-0.2522	-2.5269	-1.9186	H	0.0214	-2.4246	-1.911
H	2.7221	-2.6239	-0.7392	H	-2.1736	-1.633	-2.3644
H	2.0553	-2.0491	-2.2671	H	-2.9794	-2.0102	-0.8425
H	4.0949	-0.7946	-1.6941	H	-3.9891	-0.0386	-1.9603
H	2.7106	0.2751	-1.7462	H	-2.4276	0.7887	-1.9652
H	4.1666	-0.8075	0.7204	H	-3.5197	1.7061	-0.0763
H	0.1969	-0.7197	1.4093	H	-0.2775	-0.4561	1.3443
H	3.1541	0.1413	3.0247	H	-2.2632	2.2617	2.0417
H	2.4381	1.6185	2.3393	H	-3.0781	0.883	2.8126
H	1.4062	0.388	3.0744	H	-1.3158	1.0103	2.854
H	5.6	0.8532	-0.9827	H	-5.8548	0.6971	-0.7374
H	5.6799	2.3038	0.0339	H	-6.4686	0.0165	0.7833
H	5.9111	0.71	0.7787	H	-5.6731	1.6029	0.8
**conf 7**	X axis(Å)	Y axis(Å)	Z axis(Å)				
C	4.8448	-0.7181	0.0096				
C	5.1128	0.6178	-0.2818				
C	4.0618	1.5194	-0.4096				
C	2.7398	1.1145	-0.2334				
C	2.4643	-0.225	0.0828				
C	3.5274	-1.1384	0.1793				
O	3.3476	-2.4709	0.4416				
O	4.375	2.8105	-0.7169				
C	1.0711	-0.7041	0.2907				
C	0.045	0.2844	0.7274				
C	-1.2719	0.2349	0.4507				
C	-2.2008	1.301	0.997				
C	-3.5305	0.6999	1.4476				
C	-4.2149	-0.0443	0.294				
C	-3.2464	-0.8879	-0.5026				
C	-1.9098	-0.7659	-0.3903				
C	-3.8667	-1.8981	-1.426				
O	-4.7988	0.8951	-0.6148				
C	-6.1548	1.1886	-0.3085				
O	0.8278	-1.9018	0.1697				
H	5.6587	-1.4329	0.0965				
H	6.1378	0.9508	-0.421				
H	1.9372	1.833	-0.3602				

H	2.3841	-2.6636	0.3837				
H	3.5593	3.3231	-0.8384				
H	0.4266	1.0947	1.3418				
H	-1.7439	1.8223	1.8472				
H	-2.3774	2.0555	0.2194				
H	-3.3494	0.0031	2.2766				
H	-4.1729	1.5014	1.8288				
H	-4.9906	-0.6993	0.7131				
H	-1.27	-1.4316	-0.9655				
H	-4.4892	-2.5984	-0.8596				
H	-4.4939	-1.4	-2.1722				
H	-3.1136	-2.4837	-1.9637				
H	-6.2492	1.5958	0.7021				
H	-6.5128	1.9373	-1.0203				
H	-6.7709	0.2901	-0.4119				

The Cartesian coordinates of the lowest energy conformer for **5**							
**conf 1**	X axis(Å)	Y axis(Å)	Z axis(Å)	**conf 2**	X axis(Å)	Y axis(Å)	Z axis(Å)
C	-5.0844	-1.416	0.2209	C	-5.0785	-1.4174	0.2193
C	-3.8848	-2.0315	0.5609	C	-3.8824	-2.0352	0.5641
C	-2.6613	-1.3966	0.355	C	-2.6574	-1.3997	0.3627
C	-2.6264	-0.1133	-0.2041	C	-2.6266	-0.1134	-0.1976
C	-3.8405	0.5058	-0.5478	C	-3.8407	0.5054	-0.5445
C	-5.0593	-0.1394	-0.3371	C	-5.0581	-0.1413	-0.3376
O	-3.9173	1.7567	-1.1016	O	-3.9174	1.7565	-1.0978
O	-3.8603	-3.2803	1.1087	O	-3.9546	-3.285	1.1054
C	-1.3334	0.5982	-0.4393	C	-1.3347	0.603	-0.4324
C	1.1883	0.811	-0.3549	C	1.1884	0.8156	-0.3613
O	-1.3654	1.7191	-0.9507	O	-1.3678	1.7257	-0.9391
C	2.5319	0.0691	-0.2761	C	2.531	0.0711	-0.2878
C	2.8269	-0.5707	1.0945	C	2.8448	-0.541	1.0916
O	2.5307	-0.9622	-1.2783	O	2.5101	-0.982	-1.2669
C	5.7062	-2.7872	-0.2687	C	5.6891	-2.8046	-0.2686
C	4.4967	-1.9075	-0.1615	C	4.4865	-1.9152	-0.1615
C	3.8317	-1.5348	-1.4648	C	3.8069	-1.5617	-1.4624
C	4.024	-1.4737	1.0203	C	4.0331	-1.4556	1.0181
C	1.2323	2.001	0.5788	C	1.2372	2.0062	0.5711
O	1.8487	3.0551	-0.0238	O	1.8509	3.0597	-0.0342
O	0.8104	1.9959	1.7281	O	0.8209	1.9995	1.7225
C	1.9093	4.232	0.7826	C	1.9142	4.2375	0.7709
C	-0.0297	-0.0761	-0.0433	C	-0.0287	-0.0703	-0.042
H	-6.0393	-1.9071	0.3799	H	-6.0242	-1.9268	0.3833
H	-1.7584	-1.9274	0.6394	H	-1.7446	-1.9129	0.6428

H	-5.9866	0.3581	-0.6097	H	-5.9878	0.3509	-0.6107
H	-2.9996	2.0995	-1.1973	H	-3.0013	2.1032	-1.1902
H	-4.7734	-3.6007	1.2007	H	-3.0601	-3.605	1.3065
H	1.0669	1.1866	-1.3806	H	1.0622	1.1892	-1.3872
H	3.3331	0.7814	-0.5224	H	3.3303	0.7756	-0.5611
H	1.9859	-1.1798	1.4419	H	2.0045	-1.1345	1.4667
H	3.008	0.2102	1.8408	H	3.0453	0.2545	1.8172
H	5.4429	-3.7409	-0.7373	H	5.4135	-3.7652	-0.7154
H	6.4758	-2.3009	-0.8768	H	6.4533	-2.3347	-0.8961
H	6.1435	-3.0057	0.7112	H	6.1384	-3.0075	0.7092
H	4.4546	-0.8259	-2.0219	H	4.4247	-0.8643	-2.0393
H	3.6957	-2.4259	-2.0862	H	3.6594	-2.4625	-2.067
H	4.4923	-1.7668	1.9558	H	4.5134	-1.7333	1.9523
H	2.3872	5.0209	0.1953	H	2.3886	5.0263	0.1806
H	0.9025	4.5644	1.0545	H	0.9083	4.5691	1.0473
H	2.5136	4.0509	1.6769	H	2.5228	4.0577	1.6625
H	-0.078	-0.3075	1.0262	H	-0.0715	-0.3006	1.0279
H	0.044	-1.0097	-0.6119	H	0.0413	-1.0047	-0.6098
**conf 3**	X axis(Å)	Y axis(Å)	Z axis(Å)	**conf 4**	X axis(Å)	Y axis(Å)	Z axis(Å)
C	-4.9451	-1.559	0.3171	C	-5.242	-1.2165	0.2575
C	-3.7122	-2.1081	0.6519	C	-4.0793	-1.9718	0.3646
C	-2.5214	-1.4437	0.3638	C	-2.8289	-1.4179	0.0963
C	-2.5542	-0.1978	-0.2744	C	-2.7284	-0.0754	-0.2886
C	-3.8016	0.3538	-0.6133	C	-3.9058	0.6837	-0.4022
C	-4.9872	-0.3202	-0.3196	C	-5.152	0.1192	-0.1292
O	-3.9445	1.5637	-1.24	O	-3.9189	2.0002	-0.7818
O	-3.6217	-3.3176	1.2753	O	-4.1193	-3.284	0.734
C	-1.2984	0.5457	-0.595	C	-1.4059	0.5516	-0.5875
C	1.2169	0.8732	-0.5714	C	1.1281	0.6184	-0.536
O	-1.39	1.6219	-1.1879	O	-1.3871	1.7057	-1.02
C	2.5915	0.1977	-0.4215	C	2.3898	-0.2565	-0.624
C	2.9424	-0.1986	1.0261	C	3.7044	0.537	-0.7125
O	2.6069	-0.9836	-1.2412	O	2.4527	-1.1541	0.4908
C	5.8665	-2.5171	-0.0386	C	5.917	-2.599	-0.1487
C	4.6292	-1.6702	-0.0415	C	4.7617	-1.6442	-0.1975
C	3.9257	-1.531	-1.3703	C	3.4433	-2.1758	0.3147
C	4.1664	-1.0666	1.0675	C	4.8853	-0.3883	-0.6625
C	1.2578	2.1995	0.1649	C	1.1936	1.6013	0.6201
O	0.5184	2.1799	1.3069	O	1.7803	2.7642	0.212
O	1.9434	3.1478	-0.1994	O	0.7666	1.3951	1.7483
C	0.5206	3.4149	2.0249	C	1.8464	3.7659	1.2275
C	0.0408	-0.0388	-0.1754	C	-0.1337	-0.2518	-0.378
H	-5.8748	-2.0732	0.5398	H	-6.2177	-1.6441	0.466

H	-1.5899	-1.9238	0.6464	H	-1.9571	-2.0569	0.1956
H	-5.9414	0.1249	-0.5902	H	-6.0499	0.7251	-0.2207
H	-3.0443	1.9247	-1.4086	H	-2.99	2.2694	-0.9639
H	-4.5175	-3.6728	1.4038	H	-5.0413	-3.5298	0.9193
H	1.081	1.1193	-1.6344	H	1.0431	1.1996	-1.4642
H	3.3584	0.8874	-0.8039	H	2.2865	-0.8668	-1.5341
H	2.1301	-0.765	1.4934	H	3.8156	1.2304	0.1274
H	3.1141	0.7	1.6285	H	3.7242	1.1178	-1.641
H	6.3315	-2.5618	0.9519	H	6.1123	-2.9076	0.8833
H	5.6273	-3.5415	-0.3418	H	5.6966	-3.4913	-0.7435
H	6.6057	-2.1107	-0.7364	H	6.8358	-2.1529	-0.5437
H	4.5093	-0.8971	-2.0474	H	3.0474	-2.9309	-0.3739
H	3.8123	-2.5132	-1.8406	H	3.5826	-2.6503	1.2915
H	4.6652	-1.19	2.0246	H	5.843	-0.0044	-1.0022
H	-0.1361	3.3029	2.892	H	2.3208	4.6525	0.7979
H	1.5291	3.6468	2.3811	H	0.8411	4.0395	1.563
H	0.1324	4.2249	1.3994	H	2.4564	3.4197	2.0677
H	0.0207	-0.2052	0.9065	H	-0.1593	-0.7045	0.6195
H	0.1449	-1.0054	-0.6809	H	-0.1228	-1.0485	-1.1312
**conf 5**	X axis(Å)	Y axis(Å)	Z axis(Å)				
C	4.2277	-2.0997	0.4035				
C	2.8639	-2.3722	0.3923				
C	1.9335	-1.4003	0.0274				
C	2.3695	-0.1222	-0.3414				
C	3.7475	0.155	-0.3124				
C	4.6696	-0.8265	0.0505				
O	4.2748	1.3793	-0.6301				
O	2.3868	-3.6015	0.7405				
C	1.413	0.9502	-0.7465				
C	-0.9005	0.8997	0.1774				
O	1.8229	2.1116	-0.8132				
C	-2.1844	0.0579	0.2376				
C	-3.0748	0.138	-1.0179				
O	-1.7803	-1.3091	0.4377				
C	-5.1159	-3.0367	-0.108				
C	-4.0561	-1.9776	-0.1664				
C	-2.8859	-2.1593	0.768				
C	-4.1344	-0.926	-1.0005				
C	-1.2606	2.3677	0.2457				
O	-0.8418	2.9029	1.4251				
O	-1.8586	2.9771	-0.6306				
C	-1.0973	4.3022	1.5524				
C	-0.0339	0.6274	-1.0552				

H	4.9547	-2.8545	0.6864				
H	0.8819	-1.6689	0.054				
H	5.731	-0.592	0.0605				
H	3.5264	2.0028	-0.7721				
H	3.136	-4.1762	0.9705				
H	-0.3113	0.6508	1.0718				
H	-2.7646	0.3744	1.1169				
H	-2.4909	-6.00E-04	-1.934				
H	-3.5577	1.1187	-1.0741				
H	-5.9389	-2.8334	-0.8011				
H	-4.6935	-4.0126	-0.3681				
H	-5.5364	-3.0967	0.901				
H	-3.1886	-1.963	1.8027				
H	-2.5164	-3.1884	0.7134				
H	-4.9681	-0.8141	-1.6883				
H	-0.6964	4.6336	2.5142				
H	-0.5928	4.859	0.7565				
H	-2.1743	4.4963	1.5402				
H	-0.3285	1.2461	-1.9122				
H	-0.1146	-0.4063	-1.4037				
